# UTRGAN: learning to generate 5′ UTR sequences for optimized translation efficiency and gene expression

**DOI:** 10.1093/bioadv/vbaf134

**Published:** 2025-06-10

**Authors:** Sina Barazandeh, Furkan Ozden, Ahmet Hincer, Urartu Ozgur Safak Seker, A Ercument Cicek

**Affiliations:** Computational Biology Department, Carnegie Mellon University, Pittsburgh, PA 15213, United States; Computer Engineering Department, Bilkent University, Ankara 06800, Turkey; Department of Computer Science, Oxford University, Oxford OX1 3QG, United Kingdom; National Nanotechnology Research Center, Bilkent University, Ankara 06800, Turkey; National Nanotechnology Research Center, Bilkent University, Ankara 06800, Turkey; Computer Engineering Department, Bilkent University, Ankara 06800, Turkey

## Abstract

**Motivation:**

The 5′ untranslated region (5′ UTR) of mRNA is crucial for the molecule’s translatability and stability, making it essential for designing synthetic biological circuits for high and stable protein expression. Several UTR sequences are patented and widely used in laboratories. This paper presents UTRGAN, a Generative Adversarial Network (GAN)-based model for generating 5′ UTR sequences, coupled with an optimization procedure to ensure high expression for target gene sequences or high ribosome load and translation efficiency.

**Results:**

The model generates sequences mimicking various properties of natural UTR sequences and optimizes them to achieve (i) up to five-fold higher average predicted expression on target genes, (ii) up to two-fold higher predicted mean ribosome load, and (iii) a 34-fold higher average predicted translation efficiency compared to initial UTR sequences. UTRGAN-generated sequences also exhibit higher similarity to known regulatory motifs in regions such as internal ribosome entry sites, upstream open reading frames, G-quadruplexes, and Kozak and initiation start codon regions. *In-vitro* experiments show that the UTR sequences designed by UTRGAN result in a higher translation rate for the human TNF-α protein compared to the human Beta Globin 5′ UTR, a UTR with high production capacity.

**Availability and Implementation:**

The source code, including the model implementation and the optimization are released at http://github.com/ciceklab/UTRGAN. We downloaded the dataset from the UTRdb 2.0 database and available within the GitHub repository.

## 1 Introduction

RNA-based therapeutics necessitate both tunability and a long-lasting profile after administration to the body. To achieve this, optimization of both mRNA molecules and carriers is crucial to enhance their stability and promote tissue-specific tropism (Ulmer and Geall 2016). The level of RNA and protein expression resulting from mRNA therapeutics plays a critical role in various applications such as protein replacement therapies, genome engineering, genetic reprogramming studies, as well as vaccination and cancer immunotherapies (Rohner *et al.* 2022). Specifically, protein replacement therapies for conditions like hemophilia A and cystic fibrosis exemplify the significance of targeted mRNA expression levels. For instance, Chen *et al.* (2020) demonstrated that to attain the required factor VIII expression in mice using mRNA-laden lipid nanoparticles (LNPs), a concentration of 2 mg mRNA per kilogram of mouse body weight is necessary. Similarly, a study focusing on cystic fibrosis treatment found that a dosage of 0.1 mg/kg/day for two consecutive days was sufficient to restore the function of the CFTR gene in CFTR-knockout mice (Robinson *et al.* 2018). A phase 1 study addressing transthyretin amyloidosis utilized a dosage of 0.3 mg/kg to patients, resulting in the expression of Cas9 and the delivery of its single guide RNA to knock out the transthyretin gene, representing a potential treatment for this disease (Gillmore *et al.* 2021). Conversely, vaccination studies for viruses like Zika (Pardi *et al.* 2017), Covid (Vogel *et al.* 2021, Gebre *et al.* 2022), and influenza (Feldman *et al.* 2019), or tumor-associated antigen expression in cancer immunotherapies (Sahin *et al.* 2020), require a lower dosage of approximately 0.002–0.02 mg/kg injection to induce immunity against these viruses and tumor cells. Thus, designing stable RNA molecules and being able to control the expression levels are desirable (Andronescu *et al.* 2004, Busch and Backofen 2007, Sav *et al.* 2016).

Optimization of the 5′ UTR is a preferable approach to control the stability and expression level of the mRNA, because it has been shown to be an important region in the sequence with regard to these features (Sultana *et al.* 2020, Liang *et al.*, 2021).

Although UTR is not translated into the protein, it plays a crucial role in regulating the translation process because it contains the ribosome binding site (RBS) through which the ribosome attaches to initiate the translation. For this reason, many UTR sequences have been patented and used in the design of gene circuits (Lu *et al.* 2020, von Niessen *et al.* 2022).

The standard approach for optimizing UTR sequences is to introduce variants in existing target sequences and evaluate their effectiveness using simple algorithmic approaches. von Niessen *et al.* (2019, 2022) construct a library of 3′ UTR sequences from the human genome and identify 3′ UTR sequences that improve transcript stability (Orlandini von Niessen *et al.* 2019). Then, they generate synthetic sequences using a genetic algorithm based on sequence characteristics such as GC content, k-mer frequency, and free energy. The generated sequences are then selected with respect to their predicted translation efficiency (Cao *et al.* 2021). Similarly, Lu *et al.* (2020) identify 5′ UTRs that affect protein expression and discuss methods for pairing them. Studies also identify 3′ and 5′ UTRs for higher gene expression, and the samples are selected from naturally abundant mRNA sequences in human tissues (Burkhardt *et al.* 2022). More recently, Chu *et al.* (2024) used a predictive language model paired with random mutation for designing a library of 5′ UTRs with high translation efficiency. These models use machine learning algorithms only to predict sequence features and use those features to evaluate the generated samples. The potential of generative modeling is not fully utilized for designing and optimizing UTR sequences.

Several studies employ Generative Adversarial Networks (GANs) to generate sequences with similar characteristics to the natural DNA or RNA sequences (Linder *et al.* 2020, Wang *et al.* 2020). RNAGEN is a framework that enables the generation and optimization of synthetic piRNA sequences with some desired properties, such as binding to target proteins (Ozden *et al.* 2023). Zirmec *et al.* propose ExpressionGAN, a framework based on GANs for generating 1 kb-long regulatory DNA sequences (promoter, 5′ UTR, 3′ UTR, and terminator) (Zrimec *et al.* 2021, 2022). Using a model that predicts yeast gene expression, they optimize the full regulatory sequence considered (not just UTR) (Zrimec *et al.* 2020). The model used here allocates a relatively short and fixed length for generated regulatory regions (250 bp for 5′ UTR and 1 kb for the entire sequence), which would not fit many human genes with larger regulatory sequences, such as the MECP2 gene, where the length of the 3′ UTR extends over 8 kb (Cunningham *et al.* 2021).

In another study, Castillo-Hair and Seelig (2021) discuss the potential of using machine learning methods along with predictive models for optimizing 5′ UTR sequences. Linder *et al.* (2020) use predictive models for filtering generated *Escherichia coli* promoter sequences, and Wang *et al.* (2020) optimize DNA sequences generated using activation maximization for functional proteins. To the best of our knowledge, there is no prior work based on generative models that are tailored for generating and optimizing 5′ UTRs with respect to various metrics.

In this work, we propose UTRGAN, the first GAN-based pipeline for generating novel human 5′ UTR sequences and optimizing them to yield higher mean ribosome load (MRL), translation efficiency (TE), and higher gene expression. Our model generates 5′ UTR sequences that can be attached to any natural or synthetic gene of interest and can generate UTR sequences with variable lengths. UTRGAN optimizes the gene expression of any desired human gene solely based on the coordinates of the TSS and the 5′ UTR regions. This enables gene expression optimization (or other desired targets as described below). UTRGAN can generate 5′ UTR sequences without modifying the target DNA/RNA sequence it is attached to. Our generated sequences resemble the natural 5′ UTRs with respect to the distribution of various important characteristics, such as GC content, k-mer distance, and minimum free energy (MFE) (Trotta 2014). Furthermore, the optimization procedure enables the generation of optimized sequences for higher MRL, TE, and mRNA abundance. By using multiple sequences for optimization, we show that we can increase the predicted MRL, mRNA expression by 53% and by 61%, respectively, compared to the initial values for the designed sequences. In addition, optimization for TE results in up to a 34-fold increase in the average predicted value. Both results show that the optimization procedure works as intended. Depending on the application, our model is able to optimize the generated 5′ UTRs for a specific target DNA sequence or for a set of DNA sequences. The optimization for a single gene of interest increases the expression 2.2-fold on average and can result in up to 32 times higher expression for the best synthetic 5′ UTR. We further analyze our sequences in terms of their similarity to known regulatory motives, including internal ribosome entry site (IRES) and upstream open reading frame (uORF) sequences and demonstrate that sequences generated and optimized using UTRGAN maintain important motives and regulatory elements found in natural sequences. We also show that these motives are much less conserved in the sequences generated by other approaches, even if they have high predicted MRL values, suggesting that they might not be functional. We also conduct *in-vitro* experiments and demonstrate that UTRGAN-generated 5′ UTR sequences indeed yield a higher translation rate compared to the natural human β-globin 5′ UTR when attached to the TNF-α gene.

UTRGAN’s ability to generate and optimize 5′ UTRs for any given gene sequence will be the key enabler for the genetic circuit design. We think that UTRGAN will pave the way for mRNA-based therapeutics in the biotech industry for any application that requires gene expression control, such as cancer immunotherapy.

## 2 Methods

In this section, we discuss the model and the optimization procedure used to design 5′ UTRs. For details on experimental setup and hyperparameter optimization, refer to Notes S12 and S13, available as supplementary data at *Bioinformatics Advances* online, respectively.

### 2.1 The model

#### 2.1.1 Overall architecture

The generative model used here is a variant of a GAN (Goodfellow *et al.* 2020). The input of the GAN is a random noise sample *z*, and we train the GAN to generate realistic 5′ UTR sequences. The GAN learns to map the noise to the 5′ UTR space. Then, we can optimize the model to generate specific sequences by optimizing the input noise only. The optimization performed here does not update the weights of the generator and changes the input noise *z* instead.

The sequences generated by the GAN are scored by the selected scoring models. We use three deep convolutional neural networks to score the generated sequences. The mRNA abundance scoring model (Agarwal and Shendure 2020) predicts the log TPM expression of a given gene sequence, including a 5′ UTR. Xpresso provides three models for predicting median expression among many cell types, K562 erythroleukemia cells, and GM12878 lymphoblastoid cells (Agarwal and Shendure 2020). We use the median prediction model in this study, but the optimization can be performed for specific cell types using the mentioned models. The models used to predict the ribosome load and translation efficiency of the 5′ UTR require only the 5′ UTR sequence as input (Karollus *et al.* 2021, Zheng *et al.* 2023). We use the MTtrans 3R model that predicts TE values as a proxy for translation rate by inputting only the 5′ UTR sequence (Zheng *et al.* 2023). All models and the generator are differentiable, which enables us to optimize the input *z* with Stochastic Gradient Ascent by updating it in multiple iterations. Sequences are fine-tuned for 3000 iterations for optimizing expression and 10 000 for optimizing MRL and TE iterations and the performance in each iteration is stored. The version with the top performance is picked as the final optimized version of that sequence.

We also attempted to train a VAE for designing UTRs, as VAEs also provide us with a latent space that we can use for optimization. We were initially unable to train a VAE using one-hot encoded input sequences as UTRGAN does. The model did not converge. As a second attempt, we tried to train a VAE using byte-pair encoding (BPE) (Gage 1994) tokenized UTR sequences, but the model failed to learn the pattern of the paddings and was, in most cases, incapable of generating sequences with padding only at the end of the sequence. Our best attempts in training a VAE resulted in a model generating semantically meaningless or very biased GC content (mostly around 100% GC content) sequences.

##### 2.1.1.1 Generative model architecture

The GAN used here is based on the original Wasserstein GAN (WGAN) (Arjovsky *et al.* 2017) model that is a Convolutional GAN model (Radford *et al.* 2015) with the Wasserstein loss (Frogner *et al.* 2015) and Gradient-Penalty that improves the stability of the training (Gulrajani *et al.* 2017).

The layers of the deep learning model are modified to fit this task based on the dimensions of the data, with 128 nucleotides, each represented by a vector of length 5. The generator *G* consists of a dense layer to increase the dimension of the input noise, following five residual convolution blocks, all using the same number of channels. Finally, the output of the last residual block is fed to a convolution layer with five output channels following a softmax layer, the output of which is the one-hot encoded sequence. See Table S6 for details on the layer dimension of the generator. The input is a random vector of length 40, sampled from a normal distribution with a mean of zero and a standard deviation of 1.0. Technically called critic, the Discriminator *D* is a convolutional neural network with five residual blocks following a dense layer with one output. See Table S7 for details of the critic layers. Unlike the original DCGAN model (Radford *et al.* 2015), there is no activation function at the output of the dense layer of the critic, as the output is an unbounded score and not a probability (Arjovsky *et al.* 2017). The parameters of the critic are updated 5 times for every update performed on the generator. This means that the critic is updated 5 times more than the generator.

The initial weights of the network are sampled from a normal distribution with mean = 0 and standard deviation = 0.1. In the loss function, $L_{\mathrm{adv}}=(E_{\tilde{x}∼P_{g}}\left[D_{\tilde{x}}\right]-E_{x∼P_{r}}\left[D_{x}\right])+\lambda (E_{\hat{x}∼P_{\hat{x}}}\left[{\left(||\nabla_{\hat{x}}D(\hat{x})|{|}_{2}-1\right)}^{2}\right])$, the generator learns to minimize the distance between the distribution of the critic’s scores for the natural and generated sequences. The first part of the loss function belongs to the original WGAN model (Arjovsky *et al.* 2017), and the second part includes the gradient penalty and its coefficient λ. Here, *x* and $\tilde{x}$ are the natural and generated samples, where we sample $\hat{x}∼P_{\tilde{x}}$ uniformly along straight lines between pairs of points sampled from the data distribution Pr and the generator distribution Pg for the computation of the gradient penalty. The gradient penalty improves the training of the GAN by enforcing the Lipschitz constraint as the model proposes (Gulrajani *et al.* 2017), and this allows training for a higher number of iterations. Here, we train our model for 3000 epochs.

##### 2.1.1.2 Expression prediction optimization

The Xpresso model can predict the expression of a one-hot encoded DNA sequence with a fixed length of 10 500 nucleotides. The model receives sequences of 3500 nucleotides downstream and 7000 nucleotides upstream of a Transcription Start Site (TSS) and predicts the log TPM expression.

Let $z_{0}$ denote the initial latent vector, then the generator inputs the latent vector ($G(z_{0})$) and generates the 5′ UTR, which we call $U_{g}$. *S* denotes the gene sequence for which we design the 5′ UTR with the original 5′ UTR $U_{r}$. The expression prediction model is denoted as E(S), where *S* is the input sequence. To carry out the optimization, the initial 5′ UTRs are first generated using a fixed seed for the random latent vector. They are attached to the selected gene sequences, replacing the original 5′ UTRs, and the initial expression is measured. We define *R* as the function to replace the original 5′ UTR $U_{r}$ with the generated 5′ UTR $U_{G}$ while maintaining the length of *S* and the TSS position on the sequence ($R(U_{g},S)=S[0:7000]+U_{g}+S[7000+\mathrm{len}(U_{g}):(10500-(\mathrm{len}(U_{g})-\mathrm{len}(U_{r})))]$). The original position of the TSS in the DNA samples is the 7000th nucleotide from the start. The length of a sequence is obtained via the *len* function. We use the bracket notation to slice the sequences.

To perform the Gradient Ascent to the input latent vector for optimization, we first calculate the gradient of the output of the Xpresso model with respect to its input DNA sequences ($\frac{\partial E(R(G(z_{0}),S))}{\partial z_{0}}=\frac{\partial E(R(G(z_{0}),S))}{\partial R(G(z_{0}),S)}[7000:7000+L]\times \frac{\partial G(z_{0})}{\partial z_{0}}$) and slice the part of the gradient that corresponds to the 5′ UTR from the 7000th to up to 7128th index where the 5′ UTR is located depending on *L* which is the length of the generated 5′ UTR. In the next step, the gradient of the generator is calculated with respect to the input noise vector.

Updating the noise vector according to the gradient is supposed to increase the expression in the following iteration. This iteration is repeated 3000 times by updating the input latent vector $z_{\mathrm{new}}=z_{0}+\frac{\partial E(R(G(z_{0}),S))}{\partial z_{0}}$. We store the history of predicted expression values for each iteration and pick the best-performing sequence as the optimized version. Although the optimization is performed on the batch of sequences, the final sequences are selected independently as if the same optimization was performed separately for each element of the latent vector. This is to enable batch optimization instead of performing optimizations on individual UTRs separately.

##### 2.1.1.3 Mean ribosome load and translation efficiency prediction optimization

The FramePool model was trained on UTR sequences of lengths up to 100 bp. It utilizes the Frame-slice layer, which allows it to predict the MRL value for 5′ UTR sequences of any length (Karollus *et al.* 2021). Frame-slicing slices the sequence into 3-mers or codons, referred to as frames. It applies global pooling on the indices, which lets it work with sequences of any size. To perform the optimization with this model, again, we use the Gradient Ascent algorithm. Here, slicing is not required as the model only inputs the UTR, and the derivatives are taken directly on the 5′ UTR sequence. Considering the MRL prediction model as *M* and the initial latent vector as $z_{0}$ again, the gradient is calculated as $\frac{\partial M(G(z_{0}))}{\partial z_{0}}=\frac{\partial M(G(z_{0}))}{\partial G(z_{0})}\times \frac{\partial G(z_{0})}{\partial z_{0}}$.

We apply the gradient by updating the input vector $z_{\mathrm{new}}=z_{0}+\frac{\partial M(G(z_{0}))}{\partial z_{0}}$. This is repeated for 10 000 iterations, and then the optimized sequences are selected as done for expression maximization and explained in “Expression Prediction Optimization” section. TE optimization using MTtrans *3R* is performed similarly with a maximum of 10 000 iterations, and the best-performing sequences are selected as mentioned above. We analyze the latent space of the model on Fig. S7, available as supplementary data at *Bioinformatics Advances* online and show the effect of MRL or TE optimization in Figs S8 and S9, available as supplementary data at *Bioinformatics Advances* online.

## 3 Results

### 3.1 Overview of UTRGAN

The UTRGAN model is a deep GAN that learns to generate 5′ UTR sequences with characteristics similar to those of natural ones. This model is a variant of the WGAN-GP architecture (Gulrajani *et al.* 2017) for one-hot encoded DNA sequence. The model provides improved training over the original Convolutional GAN (DCGAN) models (Radford *et al.* 2015, Gulrajani *et al.* 2017) and is less prone to overfitting than the original WGAN architecture (Arjovsky *et al.* 2017).

**Figure 1.**
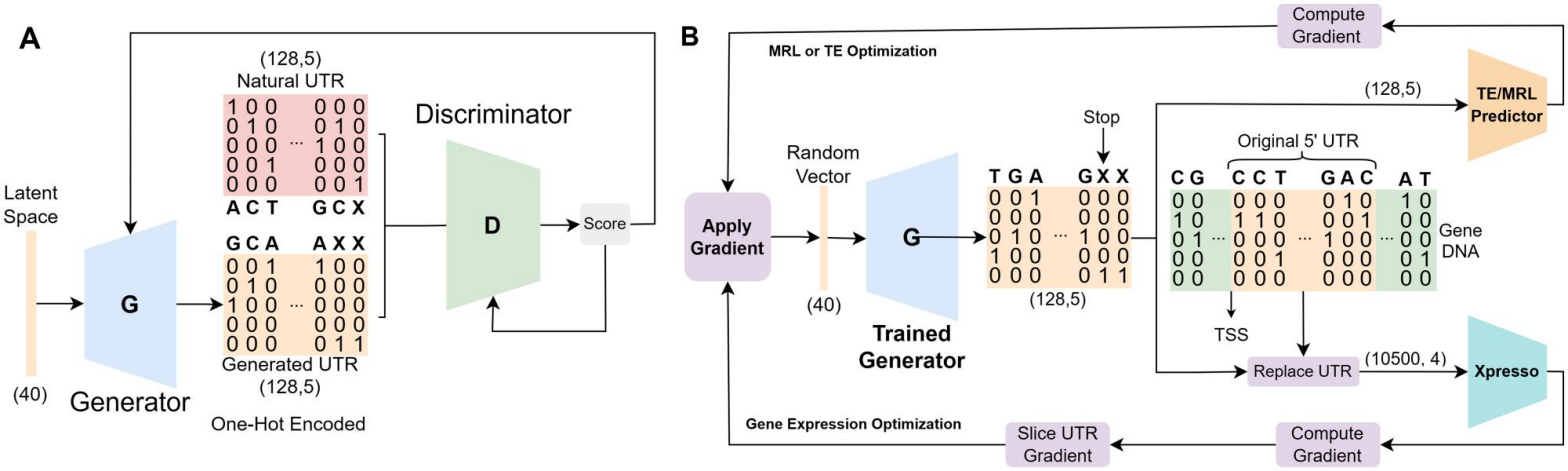
Generative and optimization architectures. Panel A shows the training phase of the GAN model. The feedback resulting from the competition between the critic and the generator updates the weights of both components. Panel B shows the optimization procedure. The GAN is used to generate samples, and the input noise is updated using the Gradient Ascent algorithm to increase the predicted MRL, TE, or mRNA abundance of the generated 5′ UTR sequences. The gene expression optimization procedure (Xpresso model) requires attaching the generated 5′ UTR to a DNA sequence to get feedback. We slice and apply the relevant gradient to update the input to generate the UTR sequence (the random vector). The MRL and TE optimization require only the UTR sequence as their input.

Figure 1A shows the overview of the architecture. The input is a noise vector. The generator and the critic are convolutional neural networks trained together. The generator upsamples the input vector using transpose-convolutions to generate 5′ UTR sequences, whereas the critic uses convolutions and dense layers to distinguish natural and synthetic 5′ UTRs. Based on this feedback, the generator learns to generate more natural-like 5′ UTRs. The optimization pipeline is shown in Fig. 1B. We use the guidance of off-the-shelf deep-learning models for optimization. To optimize the gene expression of a target RNA sequence, we use Xpresso (Agarwal and Shendure 2020), which predicts the expression of a given sequence, including the UTR region. To optimize the MRL of a UTR sequence, we use FramePool (Karollus *et al.* 2021), which predicts this value based on the UTR sequence only, independently from the RNA sequence it is attached to. Similarly, we use MTrans (Zheng *et al.* 2023) to predict the translation efficiency of a UTR sequence. We perform optimization by updating the initial input to the GAN model, which generates the UTR sequence until the generated sequence converges to its maximum predicted value independently from other sequences in its batch. That is, we apply gradient ascent on the generator’s input with respect to the feedback from the target feature predictor (MRL, translation efficiency, or gene expression).

In the following subsections, we perform various analyses to show that generated 5′ UTR sequences resemble natural 5′ UTRs and compare our performance with other approaches. The only generation tool is our approach, UTRGAN. Thus, the generated sequences are always given by UTRGAN. These generated sequences are optimized by our approach, and we call such sequences UTRGAN-optimized sequences (expression, MRL, or TE as target feature). We also compare our approach with a randomized algorithm to optimize sequences, Optimus 5-Prime. We truncate our generated sequences to the desired input length for this method, which is 50 bps, and use them as the initial input sequences for this method. We call the sequences optimized by this method Optimus 5-Prime-optimized from now on.

### 3.2 Levenshtein distances are similar in natural and generated UTRs

A metric to measure the similarity across two sets of sequences is to use the distribution of the distances to the closest sequences in a target set (i.e. natural UTR set). Levenshtein distance is the minimum number of single-character edits (insertions, deletions, or substitutions) required to change one word into the other (Levenshtein 1966). For this test, we compare the following sets: (i) UTRGAN-generated sequences (no optimization, *n* = 1204), (ii) 1024 UTRGAN-generated and MRL-optimized sequences (*n* = 1024), and (iii) sequences generated and MRL-optimized by Optimus 5-Prime (Karollus *et al.* 2021) (*n* = 1024).

We use the natural 5′ UTR dataset (*n* = 33 250) as the target set. In other words, we find the distribution of distances from the sequences in the above-mentioned sequence sets to the closest sequence in the natural UTR dataset with respect to the Levenshtein distance. This gives us three distributions, one for each of the following sequence classes: UTRGAN-generated, UTRGAN-optimized, and Optimus-5-Prime-optimized sequences. The same procedure is performed for the natural UTR set against itself to obtain the baseline distribution. There are a few natural sequences with anomalously lower distances to the natural UTR set, and we discard those in the plots.

**Figure 2.**
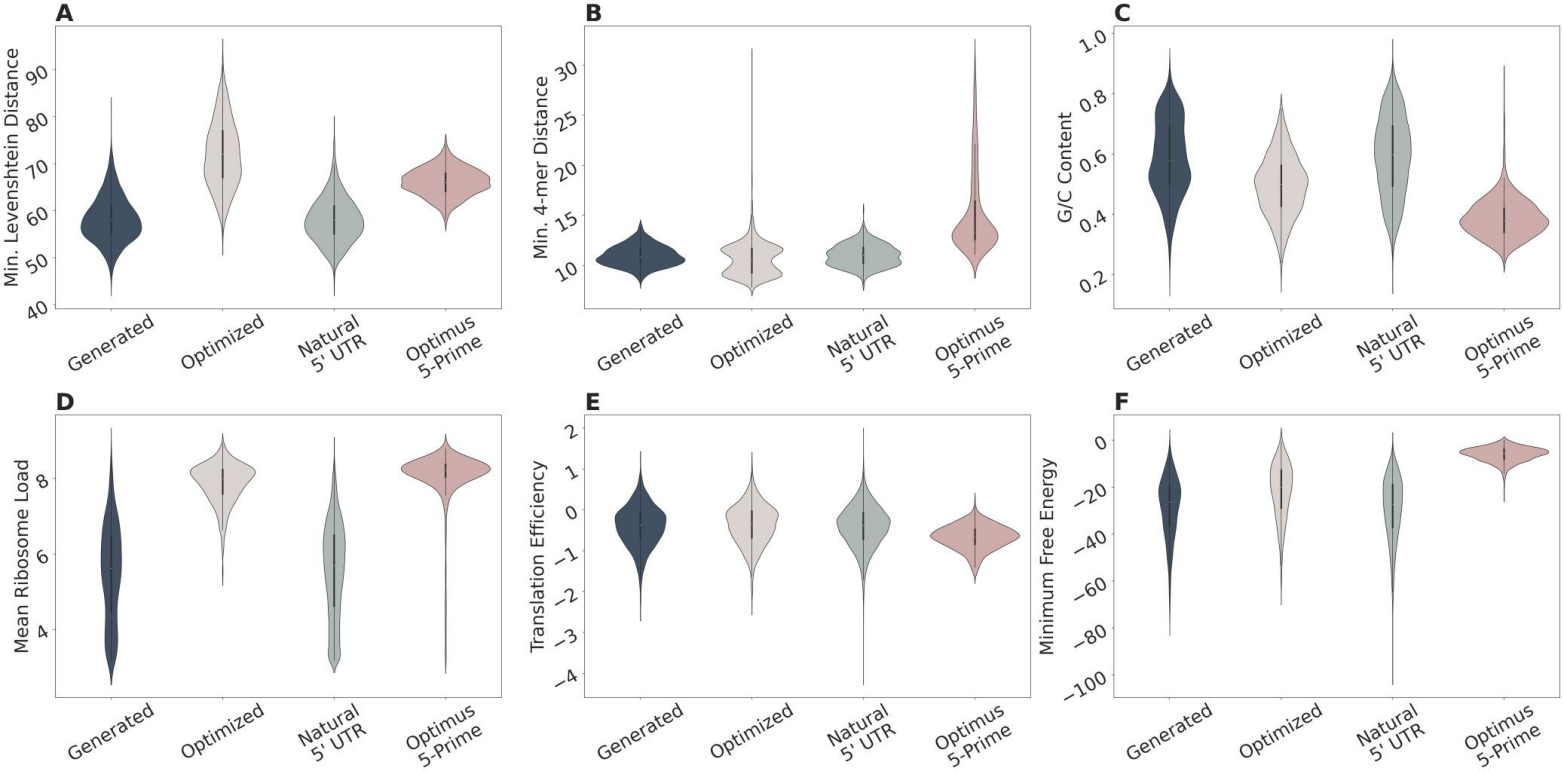
Comparison of UTRGAN-generated, natural, UTRGAN-optimized, and Optimus 5-Prime-optimized sequences. (A) The distributions of Levenshtein distances to the closest non-identical natural samples are shown for each group. (B) The distributions of the 4-mer frequency distances to the closest non-identical natural sample are shown for each group. (C) The GC content distributions are shown for each group. (D) The predicted MRL distributions are shown for each group. (E) The predicted translation efficiency distributions are shown for each group. (F) The distributions of predicted MFEs are shown for each group.

Figure 2A shows that the UTRGAN-generated sequences and the natural sequences have almost identical distributions. We also observe that the UTRGAN-optimized sequences maintain their closeness to the natural sequences while also maintaining the wide sequence length range (see Fig. S10, available as supplementary data at *Bioinformatics Advances* online). On the other hand, sequences optimized using Optimus 5-Prime have a fixed 50bps length by design and they are less diverse with respect to their distances to natural UTR sequences. The details of the statistical analysis shown in Fig. 2B–F are explained in Notes S1–S4, available as supplementary data at *Bioinformatics Advances* online, respectively.

### 3.3 Optimization yields sequences with higher expected expression

The optimization procedure, as explained in “Methods” section, is based on an iterative procedure that updates the input noise of the GAN to generate 5′ UTR sequences with higher expression. We use the model to fine-tune the sequences for higher predicted expression. The model used for prediction is a convolutional neural network-based model (Xpresso; Agarwal and Shendure 2020) that outputs the predicted log TPM expression for the input DNA sequence. The mean saliency scores in the Xpresso paper (Agarwal and Shendure 2020) are used to measure which nucleotides around the TSS affect the predicted expression more. The authors show that the most important ones are downstream of the TSS, where the 5′ UTR is placed. Based on this analysis of the Xpresso model, we expect the optimization of the 5′ UTR part of the DNA alone to increase the expression value.

**Figure 3.**
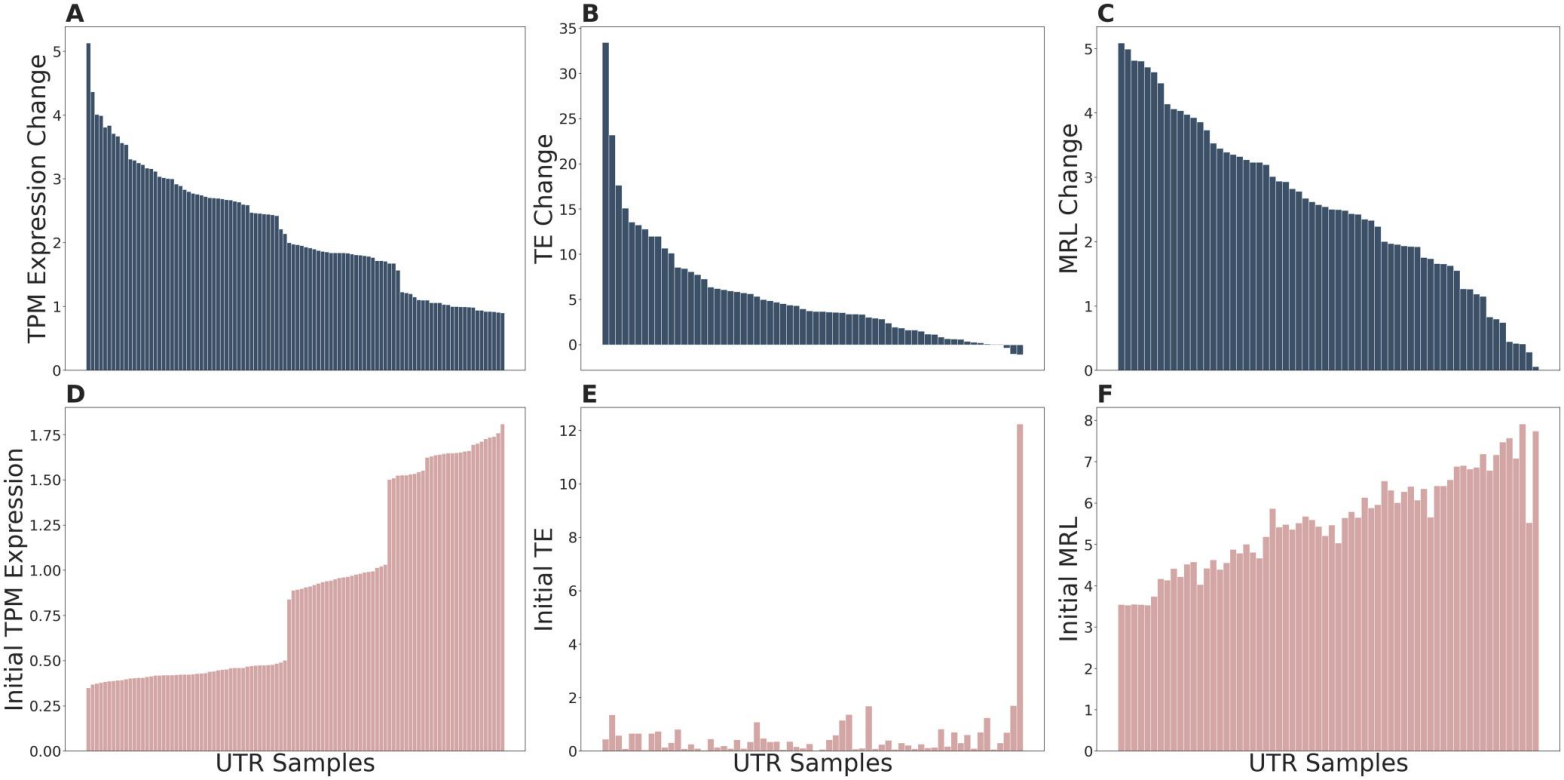
Overall performance of optimization for gene expression, TE, and MRL. We compare the average predicted values before and after optimization for each of the models. Each bar on the *x*-axis represents a 5′ UTR sequence, and the *y*-axis shows the initial values in the bottom panels and the change in the values on the top panels. (A) The gene expression change after 3000 iterations of optimization for 8 DNA samples shows that the model successfully generates 5′ UTR sequences and optimizes a majority of those to improve the original expression. The values are TPM Expression values, and the optimization yields higher predicted gene expression in 80% of the DNAs. (B) The translation efficiency change of 64 optimized generated 5′ UTR sequences is shown. In this case, a 97% majority of the sequences yield substantially higher TE after optimization. (C) Similar to gene expression and TE, optimization is effective for the majority of the sequences and performs better for sequences with low initial MRL values. (D–F) The initial gene expression, TE, and MRL values for the corresponding samples are shown in panels D, E, and F, respectively. For all three panels, initial values tend to increase towards the right. We see that it is more likely for the optimization to degrade performance for samples with high initial TE or gene expression values. Unlike gene expression and TE, degradation in the predicted MRL of the optimized 5′ UTRs is rare.

As shown in Fig. 3A, the optimization of 100 generated 5′ UTR sequences for maximizing the average expression of a set of 8 randomly selected Human genes (*MYOC*, *TIGD4*, *ATP6V1B2*, *TAGLN*, *COX7A2L*, *IFNGR2*, *TNFRSF21*, and *SETD6*) results in increased average predicted mRNA expression in 80% of the DNA samples with the optimized 5′ UTRs. To calculate a single score for each 5′ UTR, we average the expression over all 8 genes with the generated 5′ UTRs replacing the original ones. Note that we are limited by updating the 5′ UTR only, and it is not the sole determinant of the expression. Yet, we observe up to 61% increase in the average predicted gene expression compared to the initial value. In 20% of the cases, the optimization results in reduced expression compared to the initial generated sequence. In those cases, the user can pick the initially generated 5′ UTR. We discuss the possible reasons for degradation in the “Discussion” section. In addition, our optimized 5′ UTR resulted in a 38% increase in the average predicted expression of a distinct set of 8 randomly selected genes (*ANTXR2*, *NFIL3*, *UNC13D*, *DHRS2*, *RPS13*, *HBD*, *METAP1D*, and *NCALD*). This indicates that the optimization is capable of generalizing 5′ UTRs for higher average gene expression.

Furthermore, we discuss the optimization results for MRL and TE optimizations in Note S5, available as supplementary data at *Bioinformatics Advances* online (see Fig. S1, available as supplementary data at *Bioinformatics Advances* online). In addition to optimizing UTRs for multiple target genes, we optimize them for selected target genes and evaluate the performance of the model for gene-specific optimization for the *IFNG*, *TP53*, *TLR6*, *TNF*, *MYC*, *HIF1A*, *CDKN1A*, and *VEGFA* genes (see Note S6 and Figs S2 and S3, available as supplementary data at *Bioinformatics Advances* online) and compare the predicted mRNA expression of the genes using natural and gene rated sequences (see Note S7, available as supplementary data at *Bioinformatics Advances* online). Variability of generated and optimized sequences for four genes are shown in Fig. S4, available as supplementary data at *Bioinformatics Advances* online. We also perform GC content controlled optimization (see Fig. S6 and Note S6, available as supplementary data at *Bioinformatics Advances* online) and enable joint optimization of mRNA expression and TE, and show that the model successfully optimizes the two predicted values jointly (see Note S8 and Fig. S5, available as supplementary data at *Bioinformatics Advances* online). See Notes S9 and S10 and Figs S11–S13, available as supplementary data at *Bioinformatics Advances* online for motif and regulatory element analyses.

### 3.4 The cytotoxic effect of TNF-α proteins containing synthetic UTRs exhibit higher translation

Via *in-vitro* experiments, we test the efficiency of the designed UTRs. We compare the translation rate of the TNF-α protein when using (i) the UTRGAN-generated/optimized 5′ UTRs, and (ii) the human β-globin 5′ UTR. TNF-α is a pleiotropic cytokine involved in the various physiopathological processes and is known to induce cytotoxicity in select target genes, leading to cell death. The MCF-7/MX cell line is known to be vulnerable to TNF-α protein. We quantify the effect of the synthetically generated UTRs on the translation rate of this protein by using the cytotoxic effect on MCF7 cells as a proxy. See Note S11, available as supplementary data at *Bioinformatics Advances* online for the details of the experiment.

We optimize the 5′ UTR sequences with respect to the mean ribosomal load (See Methods for details). To be sure about the initiation of translation, we manually add the consensus Kozak sequence (GCCGCCACCAUGG) at the 3′ end of the 5′ UTRs (both synthetic and natural). Consequently, we design an mRNA containing the 5′ UTR region with the full-length protein sequence, encompassing the first 76 amino acids of the TNF-α protein. This specific region acts as a leader sequence for the protein’s secretion in its natural process (Wang *et al.* 1985). Subsequently, we transcribe these mRNAs *in vitro* and use them to transfect HEK293T cells, which possess a high protein production capacity and the necessary elements for secretion, such as ADAM10 and ADAM17 sheddases (Pinci *et al.* 2020).

**Figure 4.**
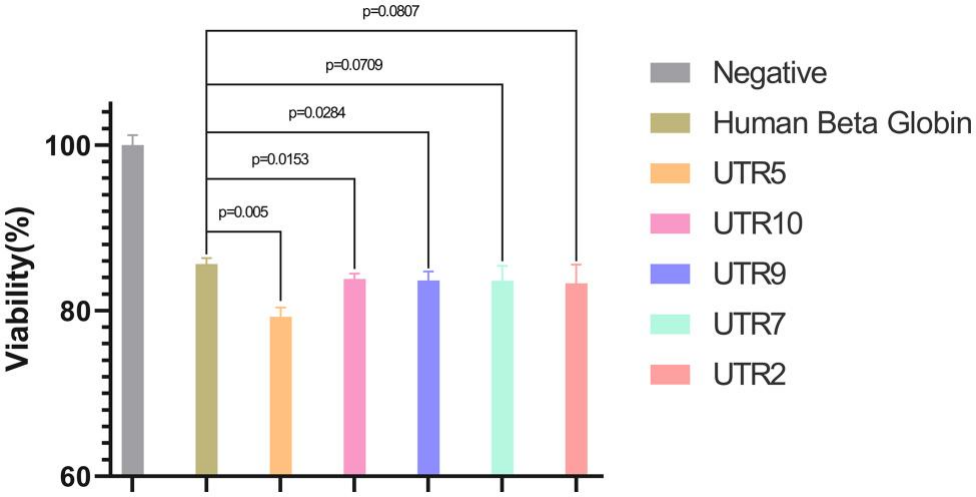
Cytotoxicity level of TNF-α with natural and UTRGAN-generated and then UTRGAN-optimized 5′ UTRs. The effects of TNF-α proteins in transfecting MCF7 cells are shown here. These values show the level of translation using different 5′ UTRs, including the human β-globin and 5 generated/optimized UTRs. The designed UTRs are more effective than the human β-globin UTR in upregulating the translation of the TNF-α mRNA.

We conduct all experiments under precisely controlled conditions for all mRNA samples, ensuring that any observed effects were solely attributable to the 5′ UTRs. Given that TNF-α induces a cytotoxic effect on MCF7 cells and inhibits their proliferation, we design an assay to quantify the TNF-α produced from given *in-vitro* transcribed mRNAs by comparing the viabilities of the MCF7 cells. According to the viability results, as shown in Fig. 4, all of the UTRGAN-generated and then optimized 5′ MRL UTRs exhibit a significantly higher cytotoxic effect than the 5′ human β-globin UTR at a 90% confidence level where three of them have a 95% confidence level with respect to the one-tailed Student’s *t*-test.

## 4 Discussion

We introduce UTRGAN, the first framework for developing synthetic 5′ UTR sequences optimized towards a target feature. Our approach enables us to generate sequences with similar attributes to the Human 5′ UTR samples without the need for exploring intractable search space of DNA sequences that have lengths of up to 128 nucleotides. We advance the state-of-the-art, which relies on optimizing existing UTRs via modifications and by generating sequences from scratch.

UTRGAN is a robust framework for synthetic 5′ UTR engineering and is applicable to numerous applications for genetic circuit design. The model’s components consist of a GAN adopted to be trained on the Human natural 5′ UTR dataset and state-of-the-art prediction models for MRL, TE, and mRNA abundance (Agarwal and Shendure 2020, Karollus *et al.* 2021, Zheng *et al.* 2023). Our model can potentially optimize sequences for given any objective function as long as a differentiable prediction model exists to predict the target metric for a 5′ UTR sequence. We can maximize and minimize the target feature.

Although UTRGAN can generate and optimize sequences with desirable characteristics, the optimization can lead to decreased TE or mRNA in less than 5% of the cases for TE in the worst optimization and 20% of the cases for mRNA abundance for optimizing UTRs for multiple genes, while it rarely happens for MRL optimization (Fig. 3C). One possible reason for decreased expression is the limitation with regard to the predictor model (Xpresso). It operates on the entire DNA (gene) sequence, and while back-propagating the gradient to update the sequence, we can only use part of the gradient that corresponds to the 5′ UTR. Thus, changing the 5′ UTR but not the rest of the sequence might lead the model to end up at a worse point in the loss space, and changing the rest of the DNA is not desirable for the target application, which requires the rest of the DNA to remain unchanged. Furthermore, in both mRNA expression and TE optimization, we observe that the decreased scores tend to occur when the optimization procedure starts with a sequence that yields a high score, as seen in Fig. 3D and E. In such a case, users can use the starting sequence and discard the optimization.

We expect to see wide use of data-driven sequences generated using deep learning methods in the near future. Generating 5′ UTRs utilizing this approach is practical and does not require massive computational resources. While we focus on 5′ UTRs, it is straightforward to generalize the framework to generate 3′ UTRs or other regulatory elements. Generating sequences with regulatory roles enables researchers to conduct experiments in a much shorter time, which can be particularly beneficial in mRNA vaccine production and mRNA-based therapeutics.

## Supplementary Material

vbaf134_Supplementary_Data

## Data Availability

The source code, including the model implementation and the optimization are released at http://github.com/ciceklab/UTRGAN. We downloaded the dataset from the UTRdb 2.0 database (Lo Giudice *et al.* 2023).
