# Supplementary material for: UTRGAN: learning to generate 5′ UTR sequences for optimized translation efficiency and gene expression
Source: Bioinform Adv. 2025 Jun 10;5(1):vbaf134. doi: 10.1093/bioadv/vbaf134 (PMC12228966; doi:10.1093/bioadv/vbaf134)
Supplement: vbaf134_Supplementary_Data [file vbaf134_supplementary_data.zip › UTRGAN Supplementary Information.pdf]

Supplementary Material
for
UTRGAN: Learning to Generate 5' UTR Sequences for Optimized Translation Efficiency and Gene Expression

1 Supplementary Notes

1.1 The generative model maintains the 4-mer distribution of the sequences

Another metric used to measure the similarity among two sets of sequences is the distribution of the k-mers within the sequences. We compute the frequency of the 4-mers for each sequence as also done in the literature Vinh, Lang, Binh, and Hoai (2015). Then, for each sequence in the natural and generated sequences, and the sequences optimized by UTRGAN and Optimus 5-Prime, we find the closest sequence (Euclidean distance) in the entire set of natural 5' UTRs, ignoring identical sequences (so natural sequences match the nearest neighbor). We obtain a distribution per group. We discard a few of the generated sequences due to their anomalously lower distances to the set of natural sequences.

We observe very similar distance distributions for the generated and natural samples as shown in Figure 1B, indicating that the generated set of sequences retain structural similarity to natural sequences and yet, are distinct in terms of the sequence, as also shown in the previous subsection. In accordance, we observe that the sequences optimized by Optimus 5-Prime have a different 4-mer distribution despite being similar to natural sequences in terms of the sequence itself.

1.2 GC content distribution of the generated 5' UTRs resemble natural 5' UTRs

The GC content of a sequence is an important aspect for the stability molecule Konu and Li (2002). We observe that the mean GC contents in the generated and natural samples are very similar, and the generated sequences are diverse with respect to their GC contents (Figure 1C). The UTRGAN-optimized sequences follow a similar distribution to that of natural sequences but have a lower GC content mean, indicating a link between GC content stability and MRL. This is in contrast to the GC content of the sequences optimized by Optimus 5-Prime, which follow a substantially different distribution.

1.3 Generated and natural samples have similar Minimum Free Energy distributions

The MFE of a 5' UTR sequence as part of the RNA sequence is another informative characteristic of the sequence regarding its stability. It depends on the number, composition, and arrangement of the nucleotides in the mRNA sequence Trotta (2014) and the 5' UTR in our case. Similar to the GC content, even though MFE might not be the sole indicator of any characteristic of a given 5' UTR sequence, we expect to see similar distributions of MFE values in a large number of samples. We calculate the MFE of the sequences we use two packages Nupack Fornace et al. (2022) and ViennaRNA Hofacker et al. (1994); Lorenz et al. (2011). The result with ViennaRNA package is presented as the results are similar.

As shown in Figure 1F, the distribution of this value is very similar in generated and natural sequences while very different in the set of the sequences optimized by Optimus 5-Prime. While the UTRGAN-optimized sequences are not exactly similar to natural sequences in the distribution of their MFE, these sequences cover a much wider range of MFE values compared to sequences optimized by Optimus 5-Prime.

1.4 Predicted Mean Ribosome Load and translation efficiency for the natural and generated sequences have similar distributions

While the above-mentioned metrics are informative about the performance of the model in generating samples structurally similar to the natural sequences, they do not reflect information on the function. MRL is a metric defined based on the ribosome count associated with an mRNA molecule and is considered a proxy for translation rate Karollus, Avsec, and Gagneur (2021). We use MRL to measure the similarity of the sequences from a functional perspective.

To estimate the MRL of a given 5' UTR sequence, we use a convolutional neural network model (FramePool) that predicts the MRL of a 5' UTR sequence Karollus et al. (2021). Figure 2D shows that the MRL distributions of the natural and the generated sequences are almost identical. We also show the predicted MRL distribution of the UTRGAN-optimized and Optimus 5-Prime-optimized sequences. As expected, both sets of sequences have a very high average MRL as they both use the FramePool Karollus et al. (2021) model to guide the optimization. However, Optimus 5-Prime fails to improve the expected MRL for all sequences, which is reflected by the very long low tail in the distribution. This method introduces random mutations in the UTR sequence, and unlike our gradient-based optimization, this may degrade the MRL for many sequences.

Similarly, we use the MTtrans model trained on ribosome profiling datasets to predict the translation efficiency of the UTR sequences Zheng et al. (2023). Translation efficiency is another proxy for the rate at which a cell translates mRNA. MTtrans is trained on various translation profiling datasets, including (i) 3 massively parallel assay (MPRA) polysome profiling datasets and (ii) 3 ribosome profiling datasets. The model trained on MPRA datasets is called *3M*, the model trained on ribosome profiling datasets is called *3R*, and the model trained on all datasets is called *3M3R*. We use the MTtrans 3R version. As shown in Figure 2E, the predicted TE for generated and UTRGAN-optimized sequences are in the same range as the natural sequences. The sequences optimized by Optimus 5-Prime, on the other hand, have a lower average predicted TE and are not distributed similarly compared to the natural sequences.

1.5 Mean Ribosome Load and Translation Efficiency increases after optimization

Unlike the mRNA abundance, the MRL is not a DNA-specific metric and does not require a target DNA or mRNA sequence for optimization. The FramePool Karollus et al. (2021) model can predict the MRL value for 5' UTR sequences of any length and does not require the UTR sequence to be attached to an mRNA sequence. We use this model and optimize the initially generated 64 5' UTRs for 10,000 iterations. The results show increased MRL in more than 95% of the generated 5' UTRs (See Supplementary Figure 1A). The number of used sequences can be increased, but we would expect similar results as each sequence is optimized separately, and the gradients are not merged. We also demonstrate in Supplementary Figure 2, that the model learns a meaningful latent space with respect to the predicted MRL values for the generated and optimized sequences.

Another model we use to optimize our generated 5' UTRs is the MTtrans model Zheng et al. (2023), which predicts translation efficiency. Translation efficiency is another proxy for the rate that a cell translates mRNA. MTtrans is trained on various translation profiling datasets, including (i) 3 massively parallel assay (MPRA) polysome profiling datasets and (ii) 3 ribosome profiling datasets. The model trained on MPRA datasets is called *3M*, the model trained on ribosome profiling datasets is called *3R*, and the model trained on all datasets is called *3M3R*. Here, we use the MTtrans *3R* model to optimize generated 5' UTRs for higher translation efficiency (TE). The results in Supplementary Figure 1B show that the optimization yields a substantial increase in TE values. To combine the optimization for mRNA abundance and translation efficiency, one can optimize many sequences for gene expression first and then select the ones also with higher MRL or translation efficiency. We also present our two-step optimization results in Supplementary Note 1.8.

In addition to the TE prediction using the 3R model, the MTtrans 3M model allows us to extract important motives from the MPRA datasets used for training the model Sample et al. (2019). It outputs a set of 256 7-mers as motives that affect the predicted MRL more than others, both positively and negatively. During MRL optimization using the FramePool model, we observed that the number of motives affecting the MRL negatively was reduced by 53% on average after optimization, while positive motives were mostly preserved or increased. This shows that optimization using our model eliminates the negative motives based on the gradients backpropagated from the MRL prediction model.

To further analyze the results of the optimization, we compare different characteristics of the generated sequences and the optimized ones for both TE (See Supplementary Figure 3) and MRL (See Supplementary Figure 4) optimizations. We see that both TE and MRL optimization favor longer UTR sequences and lower GC content. In terms of MFE, both optimizations reduce the average absolute MFE of the sequences slightly. However, there is no clear correlation between the predicted TE and MFE values in both cases.

1.6 Optimization improves the expected expression for specific target genes

As discussed above, our optimization procedure increases the gene expression when we optimize multiple generated 5' UTRs for multiple gene sequences (optimization based on average). Instead, we can work with a single target gene as well. To optimize 5' UTRs for a specific gene, we generate 64 sequences using a fixed seed and optimize them for higher expressions when placing the UTR of the target gene only.

We optimize 5' UTRs for the *TLR6*, *IFNG*, *TNF*, and the *TP53* genes from the human genome. Noreen and Arshad Noreen and Arshad (2015) discuss the role of the *TLR6* gene in the regulation of innate as well as adaptive immunity. Based on its role in the immune system, the expression level of this gene can affect the immune response. *IFNG* (*IFN-γ*) gene also has a role in the immune system and encodes an important cytokine, interferon-gamma, for immune response Benson, Liu, Deck, Mora, and Mu (2022); Sun et al. (2022). Similarly, *TNF* or *TNF-α* is the tumor necrosis factor gene and encodes cytokines that play a crucial role in the immune system's inflammatory response. Finally, *TP53* gene is one of the most important tumor suppressor genes and encodes the P53 protein Lee et al. (2020); Ozaki and Nakagawara (2011). Controlling the expression of these genes has important implications in the immunology and oncology fields. These are provided as examples to show the capacity of the model to increase the expression of specific target genes. This approach can be used on any gene for expression optimization, both to increase and decrease expression.

We show in Figure 4 that optimization for these genes improves gene expression for more than 95% of the generated 5' UTRs. It provides a 4-fold, 37%, 4.2-fold, and 63% increase in expression on average for each gene, respectively. These results show that optimization is successful on average, and users can pick the top generated sequence for their application. The performance increase for the highest expression yielding 5' UTR is 8-fold, 3-fold, 32-fold, and 3.3-fold for each respective gene. This optimization results in 2.2-fold increase in the average expression of the mentioned 4 genes together. The sequences of the best-performing 5' UTRs for these genes are provided in Supplementary Table 1.

Moreover, we observe that the GC content of the sequences increases with gene expression optimization, and the best-performing sequences exhibit GC content as high as 85 percent. The GC content of some natural sequences even exceeds this number. It should be noted that even though high GC content can lead to higher mRNA expression levels Arhondakis, Clay, and Bernardi (2008)Kudla, Lipinski, Caffin, Helwak, and Zylicz (2006), it may not be desirable in all circumstances due to various concerns such as stability Konu and Li (2002). To address this, we incorporate a control mechanism in our optimization process, allowing us to limit the maximum level of GC content for the selected best-performing sequences. For optimization results with an upper bound of 65 percent for the GC content of the optimized sequences, please refer to Supplementary Figure 1. The procedure still substantially increases the expected expression of all four genes.

1.7 Synthetic 5' UTRs yield higher predicted expression for target genes compared to their natural 5' UTRs

Our main goal in this study is not to replace existing 5' UTRs of natural genes. The experiments in the previous subsections with a single or a set of target genes are to show that the optimization mechanism can tailor the initially generated (synthetic) 5' UTR sequences toward a goal (maximize expression) using natural gene sequences as templates/examples when their UTR sequences are replaced with the generated and the optimized UTR sequences. Our approach is rather intended for novel and synthetic DNA molecules with some desired properties, such as binding to another protein after translation.

Yet, in this subsection, we investigate if the 5' UTRs we generate yield higher predicted expression compared to the natural 5' UTRs. For each of the *TLR6*, *IFNG*, *TNF*, and *TP53* genes, we generate 64 5' UTR sequences and pick the sequence that maximizes the expected expression and compare this value with the expected expression of the gene using their natural 5' UTRs. We observe that the sequences we generate yield 8-fold, 3-fold, 32-fold, and 3.3-fold increases in the expected expression, respectively.

1.8 Joint optimization results in both higher translation efficiency and gene expression

As shown in Section 2.3 and Supplementary Note 1.5, independent optimizations for translation efficiency and mRNA abundance yield positive results. Nevertheless, optimizing one does not necessarily increase the other. Here, we also consider the case where optimizing 5' UTR sequences for both higher translation rate and mRNA abundance for a target gene. We use the same set of 4 genes used in Section 1.6. We use a sequential optimization procedure to achieve this goal. Since TE optimization is gene agnostic and mRNA abundance optimization is gene-specific, we first optimize the sequences z for higher translation efficiency for 1,000 iterations. Then, we use the optimized z as the starting point for mRNA abundance optimization. The optimization first increases the average TE from negative to positive. That is, the average value is increased from 0.27 to 30.9. Then,

the TE-optimized sequence is further optimized for mRNA abundance for each of the target genes for 1,000 iterations. Although the second optimization procedure slightly decreases the average translation efficiency from 1.49 to slightly above zero for certain genes, both the translation efficiency and mRNA abundance are higher compared to the initially generated sequences, as shown in Figure 5. In addition, we observe that our two-step optimization performs better than optimizing for these two scores simultaneously. The sequences of the best-performing 5' UTRs after joint optimization for these genes are provided in Supplementary Table 2.

1.9 Regulatory Elements are Conserved in UTRGAN-generated and -optimized Sequences

One of the most important regulatory elements in 5' UTRs are uORFs, which play various roles, such as translation reinitiationBarbosa, Peixeiro, and Romão (2013); Chen and Tarn (2019); van der Horst, Filipovska, Hanson, and Smeekens (2020). We obtain the set of known human uORF sequences from uORFdb Manske et al. (2023) (n = 2,422,112) and search for human uORFs with as short as 7bp and as long as 64bp, which includes 402,140 sequences, in the set of sequences of interest. Supplementary Figure 9A shows the number of uORFs found given in each sequence set. Note that we have a larger set of natural UTRs, so the corresponding count is normalized. We observe that there are a substantial number of uORF elements present in our UTRGAN-generated/optimized sequences, while very few of them are maintained in the sequences optimized by Optimus 5-Prime. The numbers shown in Supplementary Figure 9 are the number of these elements per 1024 sequences, as all sets of sequences consist of 1024 sequences, except for the natural 5' UTRs. Therefore, we scaled the number of elements for the natural 5' UTRs to make the numbers comparable.

IRES sequences are another set of RNA elements utilized in cap-independent translation as part of the protein synthesis processShatsky, Terenin, Smirnova, and Andreev (2018). IRES sequences are most frequently found in 5' UTR regions and are used by cells to increase the translation rate of certain genesKing, Cobbold, and Willis (2010). Here, we obtain the human IRES elements from the IRESBase database Zhao et al. (2020) and compare the sequence sets of interest with respect to their maximum pairwise alignment scores to IRES elements. We use alignment as IRES sequences tend to be relatively long (longer than 128bp). As shown in Supplementary Figure 11D, UTRGAN-optimized sequences have substantially higher alignment scores compared to the generated ones and sequences optimized by Optimus 5-Prime.

We also investigate if the generated sequences are enriched with elements such as Kozak sequences and G-quadruplexes (G4) in the sequence sets of interest. G4s are elements with specific structures in G-rich regions of the 5' UTR that affect the stability of the mRNA sequenceBeaudoin and Perreault (2010); Endoh and Sugimoto (2016). We detect G4 structures using the G4Boost model Cagirici, Budak, and Sen (2022). Kozak sequences are usually located in the non-coding region upstream of the translation initiation siteYang et al. (2023). In addition to the consensus Kozak sequence (GCCGCCRCCAUGG), alternative sequences are known to play a similar regulatory role Xie et al. (2023); Xu and Zhang (2020). However, we do not see any of the Kozak sequences in many of the natural 5' UTRs, as shown in Supplementary Figure 11C. We search for exact matches of these alternative Kozak sequences with different initiation start codons ('AUG', 'AUA', 'CUG', and 'GUG') in the sequencesNakagawa, Niimura, Gojobori, Tanaka, and Miura (2008); Xu and Zhang (2020). As shown in Supplementary Figure 11A and Supplementary Figure 11B, respectively, UTRGAN-generated and optimized sequences include many instances of G-quadruplexes and Kozak sequences while in the sequences optimized using Optimus 5-Prime they rarely occur.

1.10 Motif Analyses Provide Insights on Learned Regulatory Patterns

We perform a motif analysis on the 1,024 UTRGAN-optimized sequences for *de-novo* motif identification. We use the MEME suite Bailey, Elkan, et al. (1994); Bailey, Johnson, Grant, and Noble (2015) which yields the set of motives that are enriched given a sequence set. We retrain the top 50 motives of length 7-15 bps for each sequence set of interest (UTRGAN-generated/optimized and Optimus 5-Prime-optimized). We use the same tool to identify the top 50 important motives in natural 5' UTRs, human uORF sequences, and IRES sequences. Then, we use the TomTom motif comparison tool Gupta, Stamatoyannopoulos, Bailey, and Noble (2007) to find the top-3 motif matches across these two groups (e.g., UTRGAN-optimized vs IRES). In Supplementary Figure 13, we show the top matches between the natural sequences (5' UTR, uORFs, and IRES) and UTRGAN-optimized sequences (See Supplementary Figure 12 for the same comparison for UTRGAN-generated sequences). Analyzing these motives, we observe that some motives in generated sequences seem to disappear or get modified during the optimization as the *de novo* motif identification results in different motives for generated and optimized sequences. These results show that UTRGAN successfully maintains important motives found in natural sequences, and optimization results in meaningful changes in the number of present regulatory elements for high efficiency in the desired target.

We also observe that UTRGAN-optimized sequences result in more 'U' rich motives in contrast to the initially

generated sequences that are 'A' rich instead. Studies show that 'U' rich sequences in cancerous cells are over-regulated, and there is a correlation between the percentage of 'U' and high translation in related genes Melo, de Melo Neto, and de Sá (2003); Weber et al. (2023). In line with this observation, our optimization scheme aims for patterns with higher 'U' content to achieve a higher translation rate. Additionally, we discuss the effect of TE and MRL optimization on different charactersitics of the generated UTR sequences and show the results on Supplementary Figures 8 and 9. We discuss the effect of optimization on the GC content of the UTRs in Supplementary Note 1.6 (See Supplementary Figure 6). Finally, we show that the latent space of UTRGAN separates initial 5' UTRs (low-MRL UTRs) and optimized 5' UTRs (high-MRL UTRs) meaningfully (See Supplementary Figure 5).

1.11 *In vitro* Experiments

As discussed in the results, we compare the generated 5' UTRs and the human β-globin 5' UTR in the translation of the TNF-α protein. The methodology of the conducted experiment is explained in detail in the sections below.

1.11.1 Assembling of the constructs

For the in vitro transcription (IVT) constructs, we utilized the pUC57-T7 promoter-Human -globin 5' UTR-sfGFP- Human -globin 3' UTR-118 poly(A) construct previously obtained from Genewiz for our laboratory. Five UTRs were selected randomly from a pool of top ten *de novo* UTRGAN-generated and UTRGAN-optimized UTRs based on mean ribosomal load (MRL). This pool of UTRs was designated as UTR1 to UTR10 (see Supplementary Table 4) to simplify labeling. The selected ones UTR2, UTR5, UTR7, UTR9, UTR10, and the human β-globin 5' UTR (used as a control) were cloned into the pUC57 vector as described earlier, which includes a coding region for human TNF$-\alpha$. The human TNF$-\alpha$ coding region was sourced from the pLI$-$TNF plasmid Pinci et al. (2020) (deposited to Addgene by Veit Hornung, Addgene #171179). The selected 5' UTR regions were incorporated into the coding region through polymerase chain reaction (PCR) and then ligated into the digested pUC57 vector instead of sfGFP. Following cloning, sequence verification of the constructs was conducted using Sanger sequencing (Genewiz). Throughout the cloning process, the DH5-α strain of *Escherichia coli* was employed.

1.11.2 *In vitro* transcription and removal of the inorganic contaminants

The cloned plasmids were isolated using GeneJET Plasmid midiprep kit (Thermo Scientific) from overnight-grown bacterial cultures. Linearization was achieved using a single restriction enzyme located at the end of the poly(A) tail. The linearized plasmids were purified using the Nucleic Acid Purification Kit (Monarch). To generate the IVT mRNAs, we employed the HiScribe® T7 ARCA mRNA kit (NEB). The resulting mRNAs were purified using the Monarch RNA cleanup kit (NEB). Following nanodrop analysis of the mRNAs, isopropanol precipitation Green and Sambrook (2020) was performed if any inorganic contaminants affecting the A230/260 ratio were detected. This involved mixing the mRNA samples with an appropriate amount of 3 M sodium acetate solution (pH 5.2) to achieve a final concentration of 0.3 M, followed by adding an equal volume of isopropanol. The mixture was then incubated at -20°C overnight. After incubation, the mRNAs were recovered by centrifugation at 14,000 g for 10 minutes. The resulting pellet was washed with 500 µl of ice-cold 70% ethanol and centrifuged under the same conditions. Any remaining ethanol was evaporated, and nuclease-free water was used to dissolve the mRNA samples. These samples were then stored at -80°C until the transfection experiments were conducted.

1.11.3 Cell line maintenance

We utilized the HEK293T and MCF7 mammalian cell lines for subsequent experiments. To prepare a complete growth medium for both cell lines, 440 ml of high glucose Dulbecco's Modified Eagle's Medium (DMEM) was mixed with 50 ml of heat-inactivated Fetal Bovine Serum (FBS), 5 ml of 100x L-Glutamine (200 mM), and 5 ml of 100x Penicillin/Streptomycin. This mixture was then filtered using a Corning Disposable Filter Unit with a pore size of 0.45 µm. The resulting medium was stored at +4°C. Cell media were changed every other day, and subculturing of both cell lines was performed when they reached 90% confluency.

1.11.4 mRNA transfection and sample collection

For the production of human TNF-α under different UTRs, mRNA transfection was performed on HEK293T cells. These cells were seeded into 6-well plates at a density of 300,000 cells per well, with each well containing

1 ml of growth medium. Following a 24-hour incubation period in a 37°C humidified incubator with 5% CO2, the media were aspirated, and 1 ml of fresh medium was added to each well. Subsequently, 5 µg of IVT mRNAs containing the 5' UTRs (including human β-globin, UTR2, UTR5, UTR7, UTR9, and UTR10) were transfected into the cells using Lipofectamine 3000 (Invitrogen). One well served as a negative control without mRNA to assess the impact of the transfection reagent. The transfected cells were then returned to the incubator for an additional 24 hours. After the incubation, 1.2 ml of supernatant from each well was collected into ice-chilled tubes and kept on ice. To eliminate cellular debris, all samples were centrifuged at 5800 rpm for 5 minutes. The supernatants were concentrated to 400 µl using 0.5 ml 3 kDa centrifugal filters (Amicon) and stored at +4°C for subsequent cytotoxicity experiments, which were planned to be carried out on the same day to prevent degradation.

1.11.5 TNF-α cytotoxicity assay

The cytotoxic effect of TNF-α on cancer cells Wang, Kishimoto, Bhat-Nakshatri, Crean, and Nakshatri (2005) was evaluated using the previously described methodology Ghandadi, Behravan, Abnous, Gharaee, and Mosaffa (2017). MCF7 cells were seeded into a 96-well plate at a density of 6,000 cells per well, with each well initially containing 100 µl of medium. After a 24-hour incubation period in a 37°C humidified incubator with 5% CO2, the old media were removed, and 65 µl of fresh medium was added to each well. This fresh medium was then combined with 35 µl of the concentrated TNF-α samples obtained from mRNA transfection. Top of Form All samples, including the negative control and those with different 5' UTRs (such as human β-globin, UTR2, UTR5, UTR7, UTR9, and UTR10), were studied in triplicate. After 48 hours of incubation, the supernatants were removed from the wells, and 90 µl of fresh medium was added to each well. Subsequently, 10 µl of a 5 mg/ml MTT solution was added to the wells, and the plate was incubated for 4 hours at 37°C. Following this incubation, the supernatants were discarded, and 100 µl of DMSO was added to each well to dissolve the formazan crystals. The plate, wrapped in aluminum foil, was then shaken for 15 minutes to ensure complete dissolution of the crystals. The absorbance at 570 nm was measured for each well, and the viability of each treatment was calculated by comparing it with the negative control sample.

1.12 Experimental Setup

The model is implemented using the Tensorflow 2 library and in Python. We train and optimize the model on a Super-Micro Super-Server 4029GP-TRT with 2 Intel Xeon Gold 6140 Processors (2.3 GHz, 24.75 M cache), 256 GB RAM, 6 NVIDIA GeForce RTX 2080 Ti GPUs (11 GB, 352 bit), and 2 NVIDIA TITAN RTX GPUs (24 GB, 384 bit). We only use a single TITAN RTX GPU. The training takes around 14 hours for 3,000 epochs, and we select this checkpoint based on the validation loss as well as the p-values obtained from the statistical tests. The TE, MRL, and gene expression optimization take 3, 4, and 15 minutes, respectively. Optimizing UTR sequences for higher average expression in 8 genes takes 90 minutes. GPU is not required to use the software, but reduces the execution time significantly. The main packages used to run the software are Tensorflow-gpu 2.11.0 with CUDA 11.2 compatibility and Pytorch 2.0.0. If GPU is not available, similar versions of the packages without GPU support can be used to run the optimization codes. More details on the requirements can be found on our GitHub repository at http://github.com/ciceklab/UTRGAN.

1.13 Hyperparameter Optimization

The main hyperparameters of our model include the size of the latent dimension, the number of generator layers, and the number of discriminator layers. To find the best combination of parameters, we run a grid search on a reasonable range of parameters. The model is trained up to 200,000 generator iterations. Many of the instances of the model fail to learn and overfit, resulting in the validation loss going below the training loss, and the training loss begins to increase in some cases. In the cases that the mentioned scenario doesn't happen, we select the parameters where the p-values for the statistical tests are closer to desirable values. The latent dimension in UTRGAN is 40, and we present the number of layers in Supplementary Tables 6 and 7.

2 Supplementary Figures

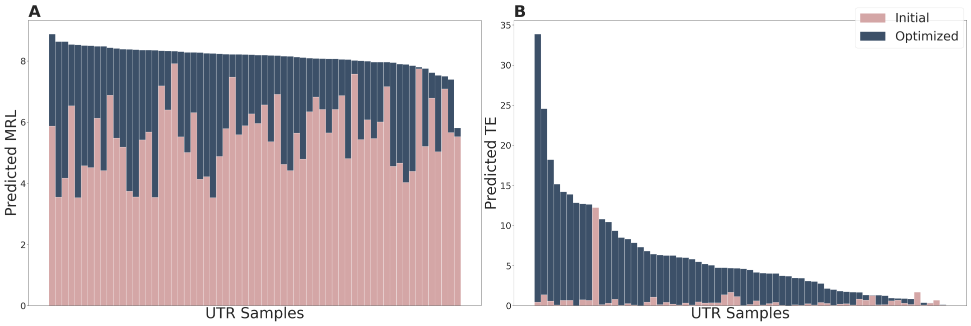

Supplementary Figure 1: **Overall performance of MRL and TE optimization. A)**. The optimization for higher MRL using FramePool results in sequences with a considerably high average MRL of 7.6. The optimization result may vary depending on the initial random sequences, and it optimizes almost all sequences to MRLs higher than 7. **B)**. Translation efficiency reaches a high value of 33 after optimization. The initial values are all around the average TE of the natural samples, and optimization increases the average more than 32-fold, and the highest optimized value is close to the maximum value of the natural sequences. The optimized values here are behind the initial values in all panels, and for the few sequences where the blue bar is not shown, the optimized value is smaller than the initial value.

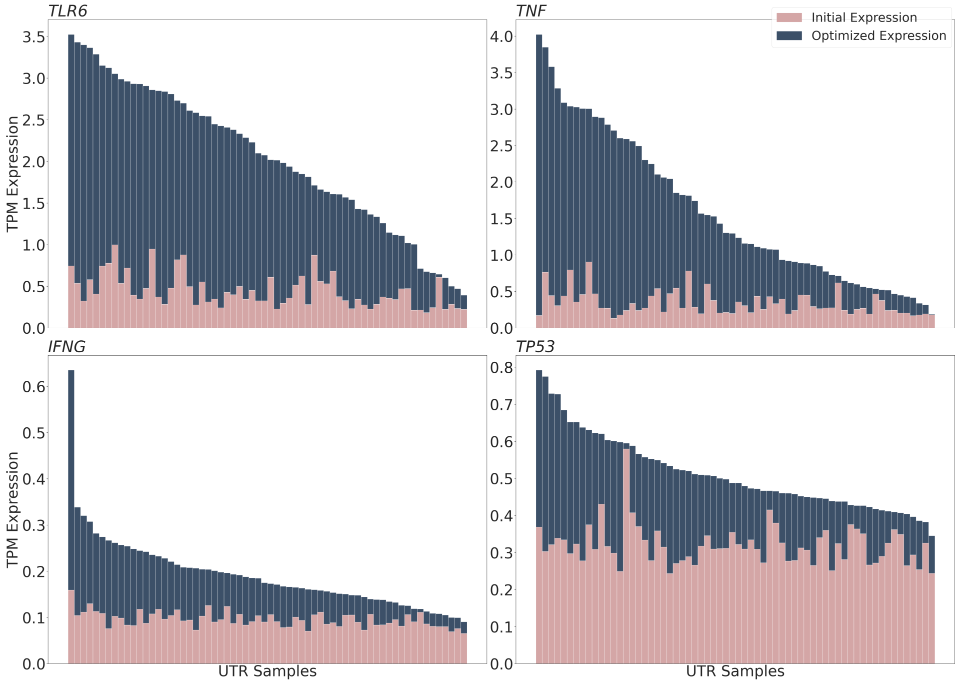

Supplementary Figure 2: **Expression optimization for specific target genes.** In addition to optimizing for random genes, it is possible to optimize many generated UTR sequences for specific genes. Here is shown the result of optimization for four specific genes. *TLR6*, *IFNG*, *TNF*, and *TP53* genes show a substantial increase in the expression for optimized sequences compared to initial predicted expression values. The optimization shows 4-fold, 37%, 63%, and 4.2-fold increase in expression on average for *TLR6*, *INFG*, *TNF*, and *TP53*, respectively. The optimized expression values are behind the initial values in all panels, and for the few sequences where the blue bar is not shown, the optimized value is smaller than the initial value.

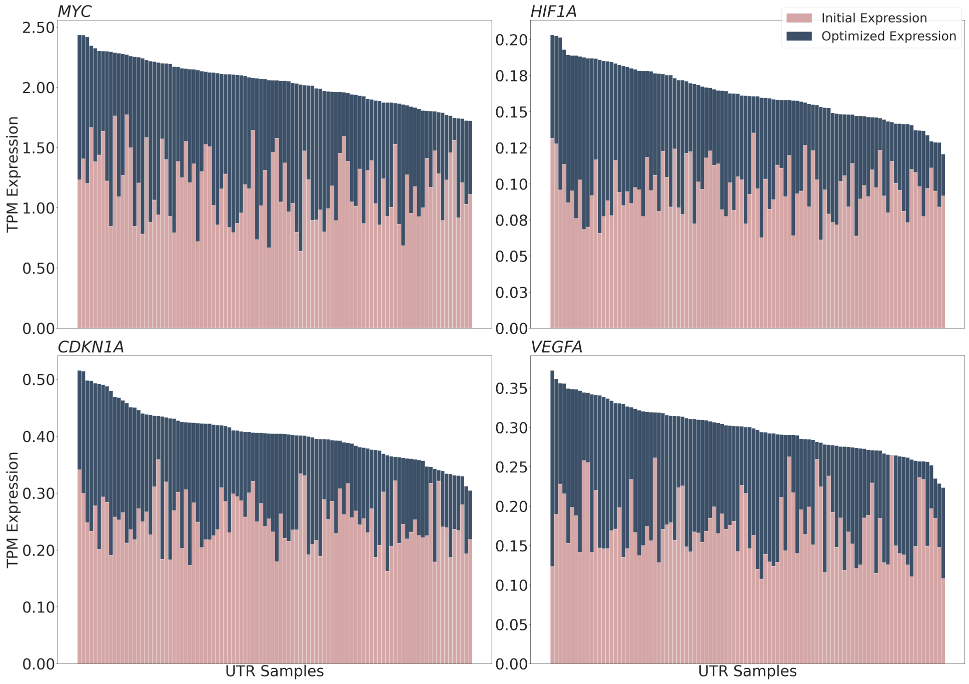

Supplementary Figure 3: **Expression optimization for specific target genes.** Here is shown the result of optimization for four specific genes. MYC, HIF1A, CDKN1A and VEGFA genes show a substantial increase in the expression for optimized sequences compared to initial predicted expression values. The optimization shows 89%, 70%, 66%, and 73% increase in expression on average for *MYC*, *HIF1A*, *CDKN1A* and *VEGFA*, respectively. The optimized expression values are behind the initial values in all panels, and for the few sequences where the blue bar is not shown, the optimized value is smaller than the initial value. The optimized 5' UTR show 2-fold, 2-fold, 80%, and 64% increase for *MYC*, *HIF1A*, *CDKN1A* and *VEGFA*, respectively, compared to the natural 5' UTRs of these genes.

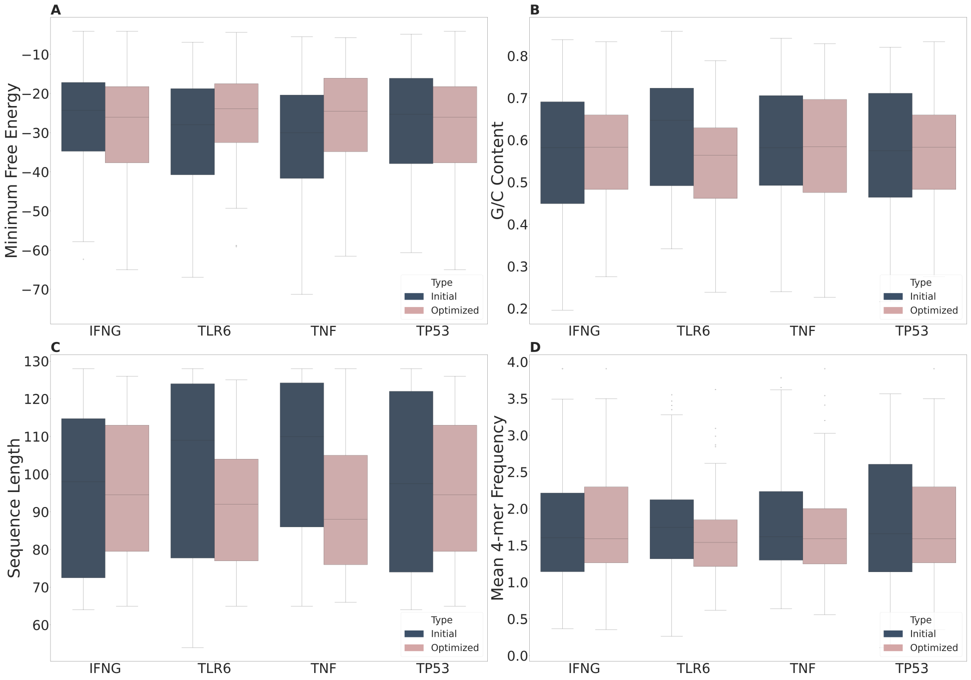

Supplementary Figure 4: **Comparison of G/C Content, sequence length, MFE, and Mean 4-mer frequency for Initial and Optimized 5' UTRs across four genes.** Panels A, B, C, and D show that both generated and optimized sequences are highly variable with respect to different sequence features.

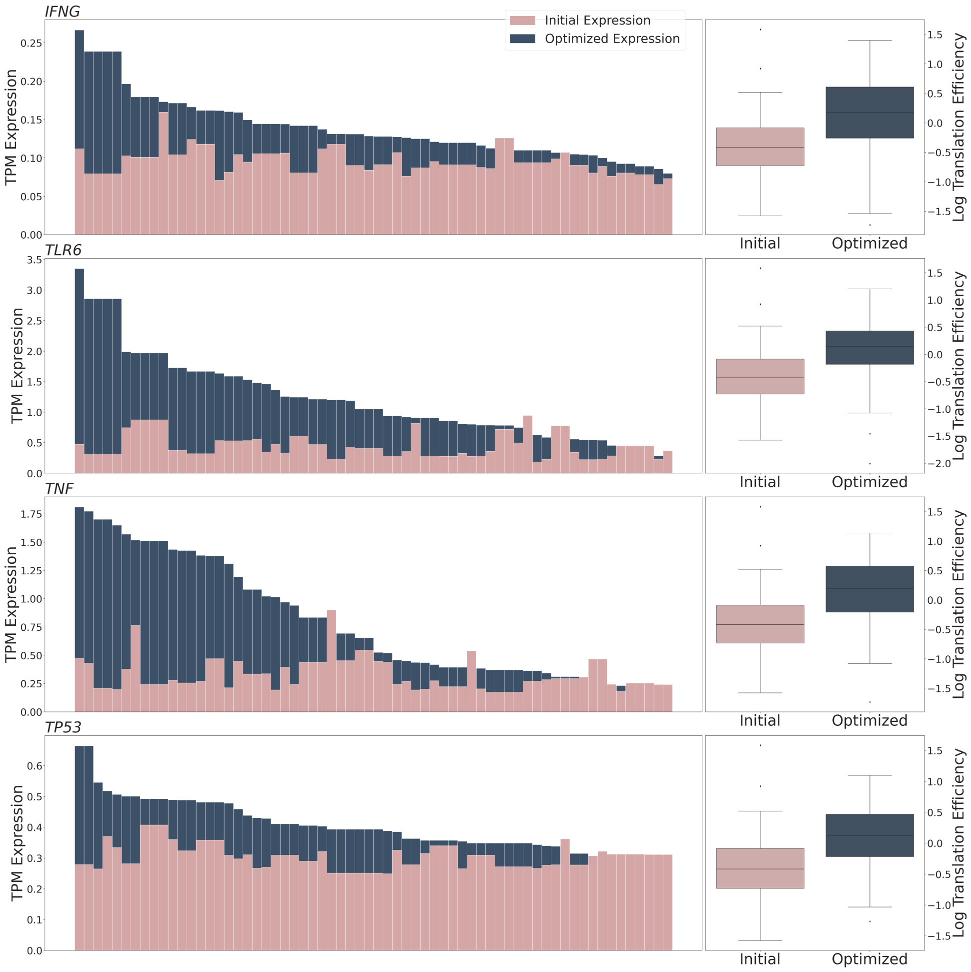

Supplementary Figure 5: **Jointly optimizing for TE and mRNA expression** The generated sequences are first optimized for high TE for 1,000 iterations, and then the sequences resulting from the optimization are used as the starting latent vector for gene expression optimization for another 1,000 iterations. As seen for the four genes, *TLR6*, *IFNG*, *TNF*, and *TP53*, the two-step optimization results in higher gene expression and translation efficiency. The predicted gene expression increases up to 12-fold, 1.8-fold, 10-fold, and 2.8-fold with respect to the natural 5' UTRs of the genes, respectively. The maximum predicted TE after optimization is at least 77% higher than the natural 5' UTR of the target genes. The optimized expression values are behind the initial values in all panels, and for the few sequences where the blue bar is not shown, the optimized value is smaller than the initial value. The box plot characterizes the TE values using the 25th, 50th, and 75th, also known as quartiles (Q1, median, Q3) and the interquartile range (IQR = Q3 Q1), with whiskers extending to a maximum of 1.5 times the IQR. Outliers beyond the whiskers are plotted separately.

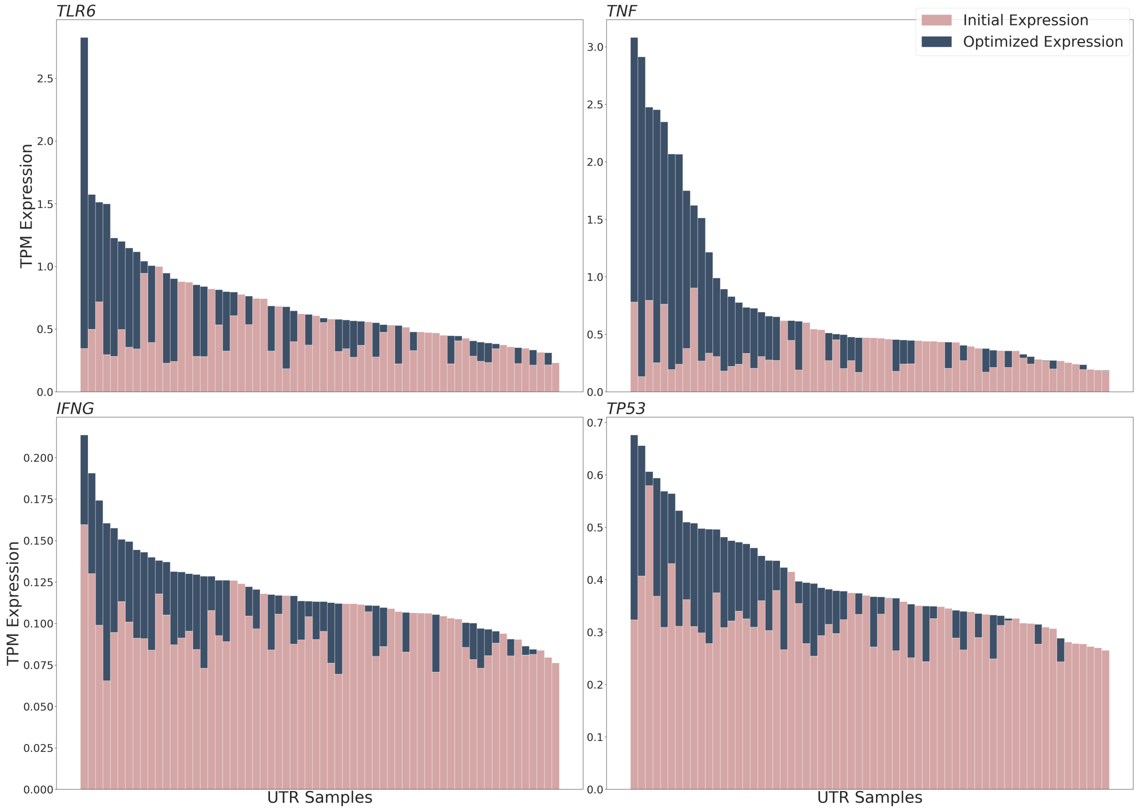

Supplementary Figure 6: **Optimization with maximum GC content limitation.** The optimized sequences with a maximum GC content limitation of 65%. We performed the optimization and selected the best sequence with the condition of their GC content being below the selected value. Although this method does not perform as well as the uncontrolled optimization in increasing the gene expression, it does result in considerable increases in all four genes (*TLR6*, *IFNG*, *TNF*, *TP53*) while maintaining the GC content. The optimized expression values are behind the initial values in all panels, and for the few sequences where the blue bar is not shown, the optimized value is smaller than the initial value.

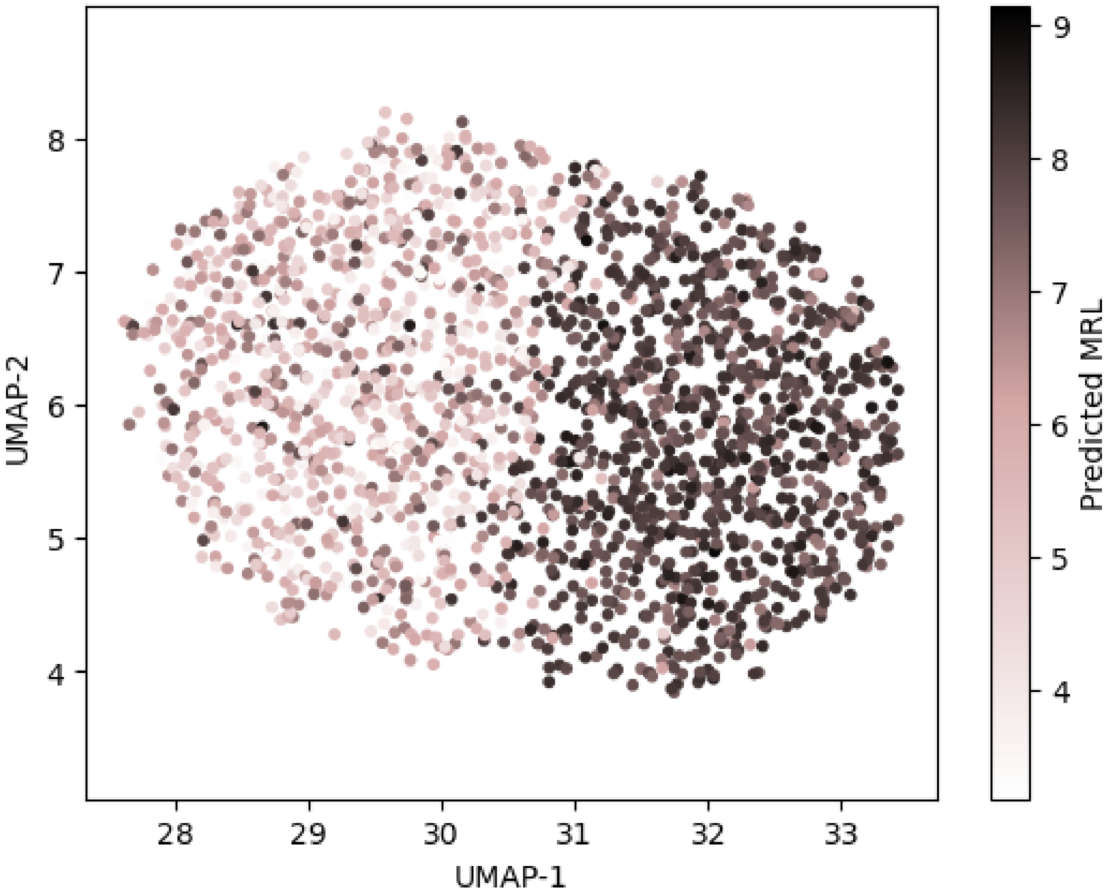

Supplementary Figure 7: **The latent space of UTRGAN for generated and optimized UTRs.** Here, we show the latent space of the model for 5' UTRs generated and optimized using UTRGAN. The UMAP is performed on the latent vector of 2048 sequences, including 1024 initially generated sequences and 1024 sequences resulting from optimization of the first set of sequences. The reduced vectors are colored by their predicted MRL values. The plot shows that the model has learned a meaningful latent space, and sequences before and after optimization are well-separated in the latent space.

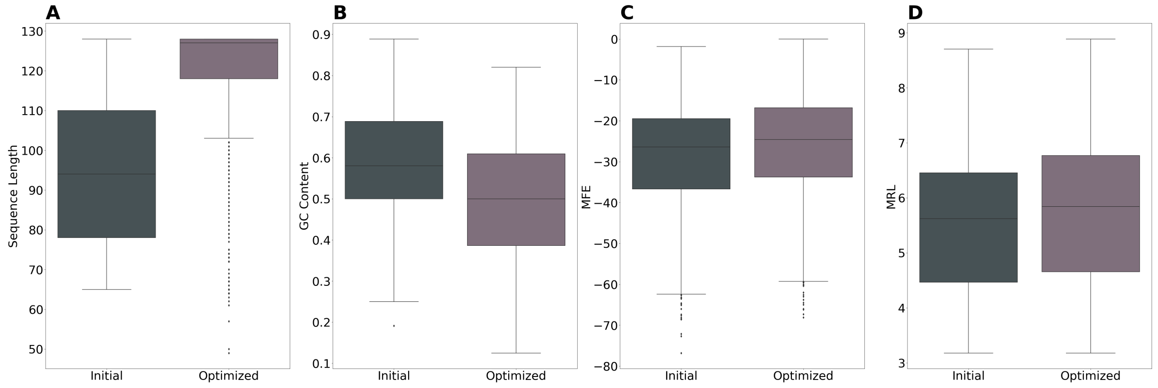

Supplementary Figure 8: **Effect of TE optimization with respect to length, GC content, MFE, and MRL of the sequences. The box plots characterize the values using the 25th, 50th, and 75th, also known as quartiles (Q1, median, Q3) and the interquartile range (IQR = Q3 - Q1), with whiskers extending to a maximum of 1.5 times the IQR. Outliers beyond the whiskers are plotted separately.**

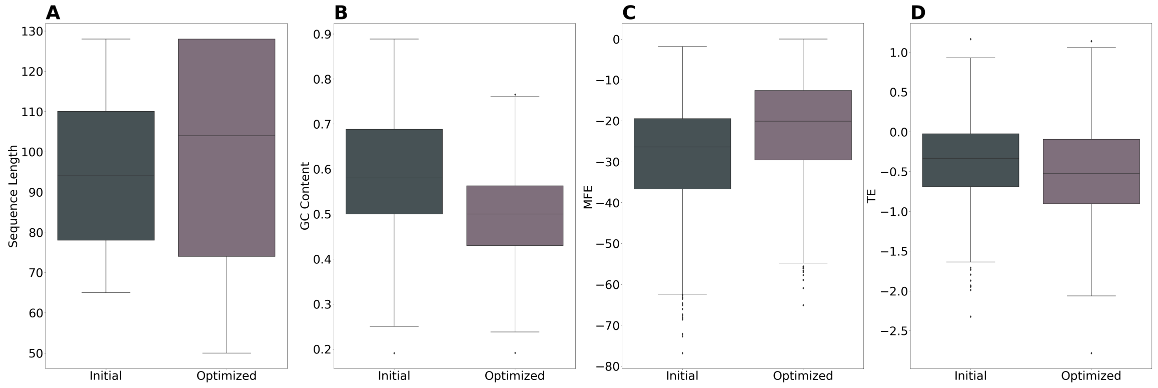

Supplementary Figure 9: **Effect of MRL optimization with respect to length, GC content, MFE, and TE of the sequences. The box plots characterize the values using the 25th, 50th, and 75th, also known as quartiles (Q1, median, Q3) and the interquartile range (IQR = Q3 - Q1), with whiskers extending to a maximum of 1.5 times the IQR. Outliers beyond the whiskers are plotted separately.**

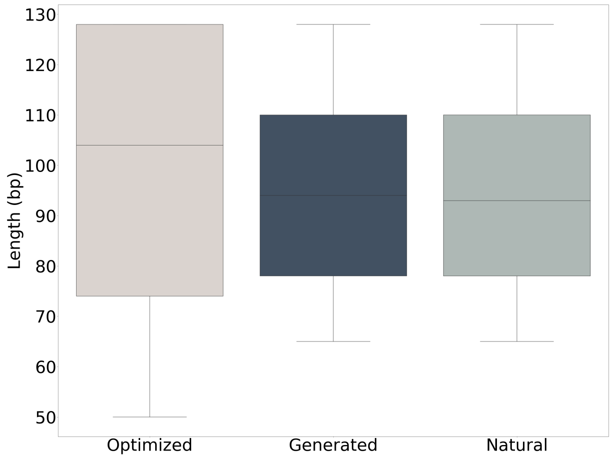

Supplementary Figure 10: **Distribution of length of the generated, MRL-optimized, and natural 5' UTRs**

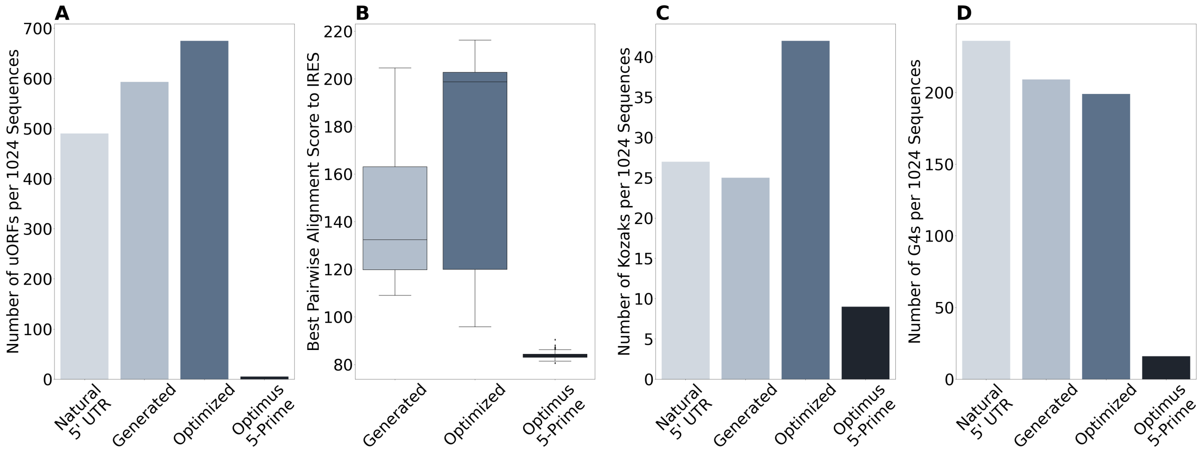

Supplementary Figure 11: **Regulatory element analysis of UTRGAN-generated, UTRGAN-optimized, Optimus 5-Prime-optimized and natural 5' UTR sequences.** The elements analyzed here include uORF, IRES, Alternative Kozak, and G4 sequences, which are shown on panels **A**,**B**,**C**, and **D**, respectively. We count the occurrence of each regulatory element in natural 5' UTR, generated, UTRGAN-optimized, and Optimus 5-Prime-optimized sequence sets. Note that all sets except the natural 5' UTR set have 1,024 sequences. So, we normalize the latter to show a number of hits for 1,024 sequences on average.

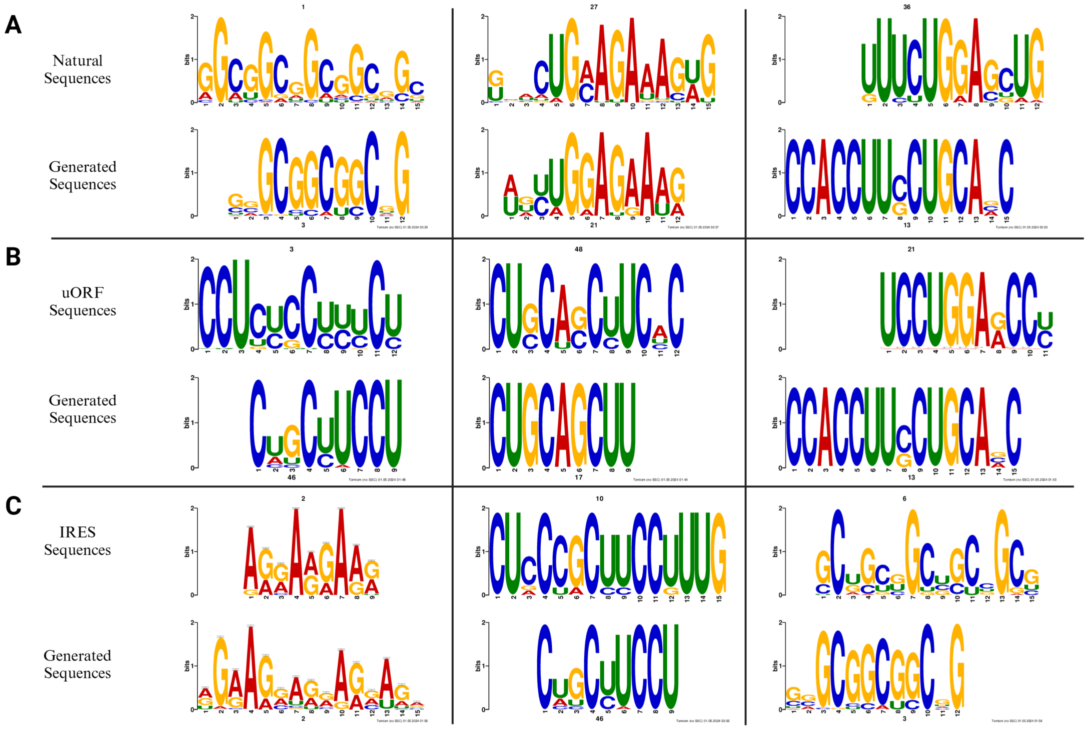

Supplementary Figure 12: **Top Matching Motives between UTRGAN-generated and various natural sequences.** The top-3 motif matches between the sequences optimized using UTRGAN and the natural 5' UTR, uORF, and IRES sequences are shown on panels **A**,**B**, and **C**, respectively. The leftmost motif is the top match for each category.

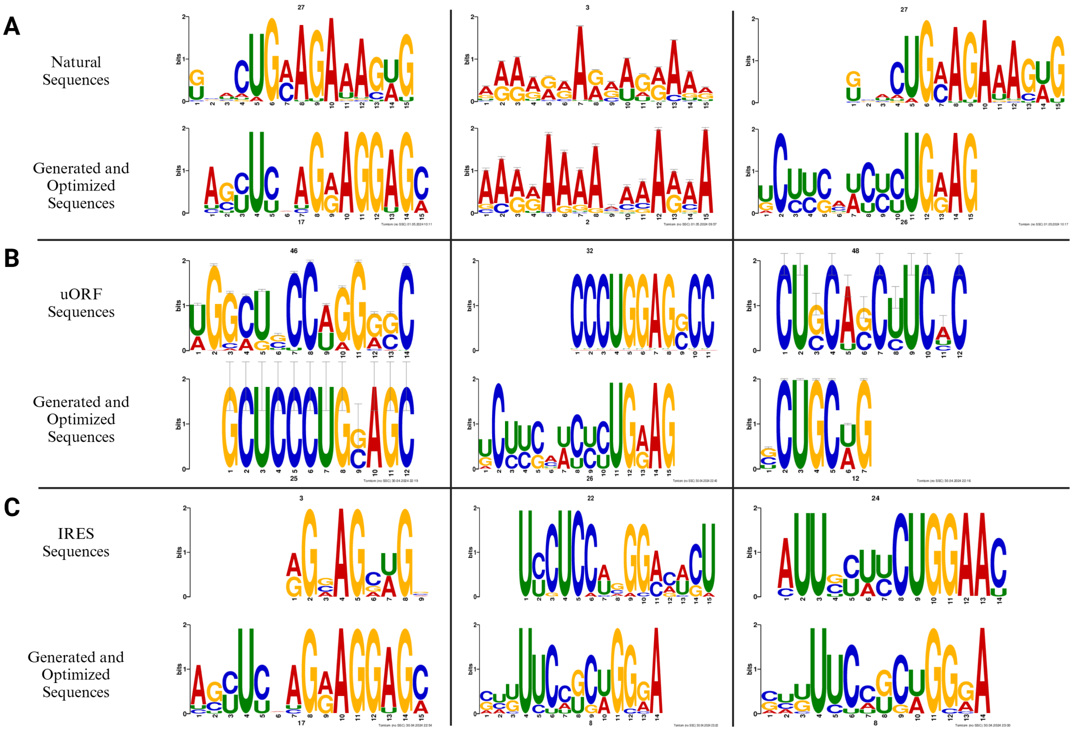

Supplementary Figure 13: **Top Matching Motives between UTRGAN-optimized and various natural sequences.** The top-3 motif matches between the sequences optimized using UTRGAN and the natural 5' UTR, uORF, and IRES sequences are shown on panels **A**,**B**, and **C**, respectively. The leftmost motif is the top match for each category.

3 Supplementary Tables

Supplementary Table 1. The top generated 5' UTR sequences for high gene expression in the target genes, that are *TLR6*, *IFNG*, *TNF*, and *TP53*. The table shows the top 5' UTR for each gene. These sequences result in higher predicted expression than the other generated ones when attached to these genes as 5' UTRs.

Gene	5' UTR
TLR6	CGGUGUCUGGAAGCCUUCUUUCGCGGUGCUGGACUCCGGGCCCGA<br>GGCCCCGAAUAGUCGCGGAAGUUCUGGGACGAAUCCGGGGCCCGG<br>UCGAACCGGCGGGGGCGCCGGCCGGCCGUCCAG
IFNG	AGACGUGGACCGCUUCGCGGCGGCUGUCGGGUGGAGUGGGAGGCG<br>AGCCGGAAGUGGCGGUCCUGGCUGCUCCGGGUCGUCCCUGCCGGC<br>GUCGGAGCGUCCCUCGGAACCAGGAGUGCCGGCGACG
TNF	GAGUUGCGGCCGGGCGGCGUCGCGGCGGUCGGGGCCGAGAGUCGU<br>GGCCGGAAGCCGCCGUUGGAUGGGGCCGGGUCGGUUGGAGACGCC<br>GGCGGCCCCGCCGCCCCCTTTGGUGGGCCCGA
TP53	CGGCUUGCUGCCGCCCGCGACAGCGGAGACAAGCAGGGAGGUCCU<br>GCCGCCUCCCAGAAAUAUUGGGGGGGCUGGGGAAGCCGCAGGCCG<br>ACUGAGUCGGGAGGCGGACGGCGGAAGGACGCGGCGGA

Supplementary Table 2. The top generated 5' UTR sequences for joint optimization of translation efficiency and high gene expression in the target genes, that are *TLR6*, *IFNG*, *TNF*, and *TP53*. The table shows the top 5' UTR for each gene. These sequences result in higher predicted expression as well as higher predicted translation efficiency than the other generated ones when attached to these genes as 5' UTRs.

Gene	5' UTR
TLR6	UGGCUGUGGCGGCCGGAAAUCUGAGCCUCCGGGCCAGUGGGCCGGG<br>AGCGGUCGGGUCUGGAGCGGCGGAGGGAGAACGGAAGCUGCGGAGG<br>CCCCGCACGCGGGGGGCUGAGGUCGGCCUCGUCAGU
IFNG	UGGCUGUGGCGGCAGGAAUUCUGUGCCUCCGGGCCUGUGGGUCGGG<br>AGCGGCCGGGUCUGGAGCGGCGGUGGGAGAAAGGAAGCGGCGGUGG<br>CCCCGCAAGCGGGGGGCUGAGGGCGGCCGGGGCGG
TNF	AGUCCGCUGGCGUGGCCCAGCCCCGGGGCCGGUUCUCGGGAACGAG<br>AAGCUGGUGAUCUCCGUUGGGGCGCGGUCGGAUGCGGUUGAGGGAG<br>GCGAGGACGCGAAAGCGGGCCCGCAGCGGAGCCGGC
TP53	GGGAGGUGGUUCCGAAAUCGCCGUGAAGCCAAGCCCUCUGCUCCGU<br>UUCCUCUUUUCCACGGACUGGGAGGUUUUCGGGACCACGCCAGGAG<br>AACCACGAGGGCUAGGCAGAGGACUGAAGCGUCGGA

Supplementary Table 3. The top generated 5’ UTR sequences for GC content controlled high gene expression in the target genes, that are *TLR6*, *IFNG*, *TNF*, and *TP53*. The table shows the top 5’ UTR for each gene. These sequences result in higher predicted expression compared to other generated 5’ UTRs with GC content lower than 65%.

Gene	5’ UTR
TLR6	GUGAAAGGCGUGCCGCCGCCCUAGAGUCCUGGGAAGACAGUCAAG<br>CGGAGAGAGGGAAGUUUCCCUGUACUUCCUCCCUCCCGGCCGCGAGU<br>CCUGGCAUCCCGCUGAGGCUGG
IFNG	UGGCUGUGGCGGCAGGAAUUCUGUGCCUCCGGGCCUGUGGGUCGGG<br>AGCGGCCGGGUCUGGAGCGGCGGUGGGAGAAAGGAAGCGGCGGUGG<br>CCCCGCAAGCGGGGGGCUGAGGGCGGCCGGGGCGG
TNF	CUUCGCCCUUCUUCAUAAUCAGUCGACAAGCGGUUUCUGGAAGGCG<br>GCGUCUCGCUGUUGCUGUGGCCUCUGGAAGCAGGAGAAGAGGCGGC<br>UUGGAAACGCUGGAAGCGGCCGCGGCGUCCUCAG
TP53	AAGACGAGGCCAGGCUGCGCAAAUCCGGCUGGGGCCGAGUUCUUGG<br>UCCGUUUUCCCAAUAAGAGUGGUCCCGGGAAUGGACGAGACGCCGC<br>CGGAAGCGUCUCUGGAGGUUGAUGGCAAG

Supplementary Table 4. The top 10 generated 5' UTR sequences for optimized MRL.

UTR	Predicted MRL	UTR
GAGGAGCAGAAAUUGGCCGCUGUUGCGUUAAUCCACAACGGUGCUGUUG UGAGUUGACAGCAGGUCCAGGGAUAAGGUGUGGUGAAGUUGGUGUCUGUAG CAUAUCCAUGGAGAUGAUUGCAAUAGUA	9.06	UTR1
UUUAACACAUAUCAGAAAAAGAAUCAAGAACUGUUGGAGCCGGGUUAAAA AAAACUUUCCAAGUAAUGUUAAAG	9.02	UTR2
CUUUCCUGAGAAUUCCCUGGAAGCACUUGUUCAACCACAGCACUAGGUUUC AAAGCAAGGUUCACACCCAGCCUCCACAAGGCCUGGGGAUGAUGAAGAGG GGAAUA	8.91	UTR3
GUUUUAUCCAGCAUCCAUUCGAGAACUUGAUCAAGUCCCACAUGCUACCCAG GACUAGGAGAGAAGGAAGCAGAAAGACAGUAAAUUGCCGCGGAUAUUUUCAG	8.81	UTR4
GUUACCAUACUGUAAUCAGAAUUCUGCUUCUAAGGAUAAAUUUACUUCUACU UAUUAUAAAAAAAG	8.80	UTR5
UGGACUCAGUCCCAACCAAAUAGUGGUGUUUCUGCAGCUCCAAACUUGAGAGA AGAACUUCAAGCUCUCCUUUAAACCGGUGUUUGC	8.74	UTR6
GUGUUUCCCAUUUAGUCACUAAGAGGUGACGCCAUACCACAUACAGGUGGAGG AGCAUUAAAGC	8.577	UTR7
GGGUGGUCGGAGCGGCUGCAGGAGGCAGUAGGAAGAAGAGAAAAGGAAGGAGG AGGAUCCGAACACGUCACAAAACAGUCGG	8.575	UTR8
AGAAACCAGAAGACCUUCUGGAGGAAGACUUC CAUAGCUUUGUCUCAGUGAAGGCAGC	8.56	UTR9
AGAAAUUUCAGUAAUAAUCGUCCUGGAGGAGUGGGUAACAUAUUUGAAGAUUUC CAUUUCAGCUUU	8.49	UTR10

Supplementary Table 5. The top 3 generated 5' UTR sequences for optimized translation efficiency. The predicted *log*-TE for these sequences is 1.5, 1.3, and 1.2, respectively.

GAAUCUCAUCUUCGCCCUUAACCAUCUCAGAGACCUUCCAACAGAA<br>CUUGGUGUAAAAGCCAGGCUUCCCCGCCUCUGCAGUGCUGCGGCUU<br>GGCUGCCUCUCCGCGCGAGG
GACCAAGCACACCUCACCCCGCGCCCCCAGUCCGGGCGCCGGGCUC<br>CUCGGUGGUCUCAGCUGCU
ACCAUCCCUCCAGCAGCUGGGACGUGCCUGCGCCGCAGCCGUGGCC<br>GCCUCGCUGCGGCUGUCUCCCCCCAG

Supplementary Table 6. The layers of the Generator in the WGAN model we use to generate realistic 5' UTR sequences. In this configuration, the batch size for training the model is 64, as seen in the table.

Input/Layer	Kernel size	Output shape
z_0	-	64 x 40
Dense	-	64 x 5120
Reshape	-	64 x 128 x 40
Convolution (1D)	1	64 x 128 x 40
Residual block (1D)	[5]x2	64 x 128 x 40
Residual block (1D)	[5]x2	64 x 128 x 40
Residual block (1D)	[5]x2	64 x 128 x 40
Residual block (1D)	[5]x2	64 x 128 x 40
Residual block (1D)	[5]x2	64 x 128 x 40
Convolution (1D)	1	64 x 128 x 5
Softmax	-	64 x 128 x 5

Supplementary Table 7. The layers of the Discriminator (Critic) in the WGAN model we use to generate realistic 5' UTR sequences.

Input/Layer	Kernel size	Output shape
Convolution (1D)	1	64 x 128 x 40
Residual block (1D)	[5]x2	64 x 128 x 40
Residual block (1D)	[5]x2	64 x 128 x 40
Residual block (1D)	[5]x2	64 x 128 x 40
Residual block (1D)	[5]x2	64 x 128 x 40
Residual block (1D)	[5]x2	64 x 128 x 40
Flatten	-	64 x 5120
Dense	-	64 x 1

References

Arhondakis, S., Clay, O., & Bernardi, G. (2008). Gc level and expression of human coding sequences. *Biochemical and Biophysical Research Communications*, *367*(3), 542–545.

Bailey, T. L., Elkan, C., et al. (1994). Fitting a mixture model by expectation maximization to discover motifs in bipolymers.

Bailey, T. L., Johnson, J., Grant, C. E., & Noble, W. S. (2015). The meme suite. *Nucleic acids research*, *43*(W1), W39–W49.

Barbosa, C., Peixeiro, I., & Romão, L. (2013). Gene expression regulation by upstream open reading frames and human disease. *PLoS genetics*, *9*(8), e1003529.

Beaudoin, J.-D., & Perreault, J.-P. (2010). 5-utr g-quadruplex structures acting as translational repressors. *Nucleic acids research*, *38*(20), 7022–7036.

Benson, L. N., Liu, Y., Deck, K., Mora, C., & Mu, S. (2022). Ifn-γ contributes to the immune mechanisms of hypertension. *Kidney360*, *3*(12), 2164–2173.

Cagirici, H. B., Budak, H., & Sen, T. Z. (2022). G4boost: a machine learning-based tool for quadruplex identification and stability prediction. *BMC bioinformatics*, *23*(1), 240.

Chen, H.-H., & Tarn, W.-Y. (2019). uorf-mediated translational control: recently elucidated mechanisms and implications in cancer. *RNA biology*, *16*(10), 1327–1338.

Endoh, T., & Sugimoto, N. (2016). Mechanical insights into ribosomal progression overcoming rna g-quadruplex from periodical translation suppression in cells. *Scientific reports*, *6*(1), 22719.

Fornace, M. E., Huang, J., Newman, C. T., Porubsky, N. J., Pierce, M. B., & Pierce, N. A. (2022). Nupack: Analysis and design of nucleic acid structures, devices, and systems.

Ghandadi, M., Behravan, J., Abnous, K., Gharaee, M. E., & Mosaffa, F. (2017). Tnf-α exerts cytotoxic effects on multidrug resistant breast cancer mcf-7/mx cells via a non-apoptotic death pathway. *Cytokine*, *97*, 167–174.

Green, M. R., & Sambrook, J. (2020). Precipitation of rna with ethanol. *Cold Spring Harbor Protocols*.

Gupta, S., Stamatoyannopoulos, J. A., Bailey, T. L., & Noble, W. S. (2007). Quantifying similarity between motifs. *Genome biology*, *8*, 1–9.

Hofacker, I. L., Fontana, W., Stadler, P. F., Bonhoeffer, L. S., Tacker, M., Schuster, P., et al. (1994). Fast folding and comparison of rna secondary structures. *Monatshefte fur chemie*, *125*, 167–167.

Karollus, A., Avsec, Ž., & Gagneur, J. (2021). Predicting mean ribosome load for 5'utr of any length using deep learning. *PLoS computational biology*, *17*(5), e1008982.

King, H. A., Cobbold, L. C., & Willis, A. E. (2010). *The role of ires trans-acting factors in regulating translation initiation.* Portland Press Ltd.

Konu, Ö., & Li, M. D. (2002). Correlations between mrna expression levels and gc contents of coding and untranslated regions of genes in rodents. *Journal of Molecular Evolution*, *54*(1), 35–41.

Kudla, G., Lipinski, L., Caffin, F., Helwak, A., & Zylicz, M. (2006). High guanine and cytosine content increases mrna levels in mammalian cells. *PLoS biology*, *4*(6), e180.

Lee, Y.-J., Seo, H. W., Baek, J.-H., Lim, S. H., Hwang, S.-G., & Kim, E. H. (2020). Gene expression profiling of glioblastoma cell lines depending on tp53 status after tumor-treating fields (ttfields) treatment. *Scientific reports*, *10*(1), 1–14.

Lorenz, R., Bernhart, S. H., Höner zu Siederdissen, C., Tafer, H., Flamm, C., Stadler, P. F., & Hofacker, I. L. (2011). Viennarna package 2.0. *Algorithms for molecular biology*, *6*, 1–14.

Manske, F., Ogoniak, L., Jürgens, L., Grundmann, N., Makałowski, W., & Wethmar, K. (2023). The new uorfdb: integrating literature, sequence, and variation data in a central hub for uorf research. *Nucleic Acids Research*, *51*(D1), D328–D336.

Melo, E. O., de Melo Neto, O. P., & de Sá, C. M. (2003). Adenosine-rich elements present in the 5-untranslated region of pabp mrna can selectively reduce the abundance and translation of cat mrnas in vivo. *FEBS letters*, *546*(2-3), 329–334.

Nakagawa, S., Niimura, Y., Gojobori, T., Tanaka, H., & Miura, K.-i. (2008). Diversity of preferred nucleotide sequences around the translation initiation codon in eukaryote genomes. *Nucleic acids research*, *36*(3), 861–871.

Noreen, M., & Arshad, M. (2015). Association of tlr1, tlr2, tlr4, tlr6, and tirap polymorphisms with disease susceptibility. *Immunologic Research*, *62*(2), 234–252.

Ozaki, T., & Nakagawara, A. (2011). Role of p53 in cell death and human cancers. *Cancers*, *3*(1), 994–1013.

Pinci, F., Gaidt, M. M., Jung, C., Kuut, G., Jackson, M. A., Bauernfried, S., & Hornung, V. (2020). C-tag tnf: A reporter system to study tnf shedding. *Journal of Biological Chemistry*, *295*(52), 18065–18075.

Sample, P. J., Wang, B., Reid, D. W., Presnyak, V., McFadyen, I. J., Morris, D. R., & Seelig, G. (2019). Human 5

utr design and variant effect prediction from a massively parallel translation assay. *Nature biotechnology*, *37*(7), 803–809.

Shatsky, I. N., Terenin, I. M., Smirnova, V. V., & Andreev, D. E. (2018). Cap-independent translation: what's in a name? *Trends in biochemical sciences*, *43*(11), 882–895.

Sun, Y., Hu, B., Stanley, G., Harris, Z. M., Gautam, S., Homer, R., … Rajagopalan, G. (2022). Interferon-γ is protective in cytokine release syndrome-associated extrapulmonary acute lung injury. *American Journal of Respiratory Cell and Molecular Biology*.

Trotta, E. (2014). On the normalization of the minimum free energy of rnas by sequence length. *PloS one*, *9*(11), e113380.

van der Horst, S., Filipovska, T., Hanson, J., & Smeekens, S. (2020). Metabolite control of translation by conserved peptide uorfs: the ribosome as a metabolite multisensor. *Plant physiology*, *182*(1), 110–122.

Vinh, L. V., Lang, T. V., Binh, L. T., & Hoai, T. V. (2015). A two-phase binning algorithm using l-mer frequency on groups of non-overlapping reads. *Algorithms for Molecular Biology*, *10*(1), 1–12.

Wang, Z., Kishimoto, H., Bhat-Nakshatri, P., Crean, C., & Nakshatri, H. (2005). Tnf resistance in mcf-7 breast cancer cells is associated with altered subcellular localization of p21cip1 and p27kip1. *Cell Death Differentiation*, *12*, 98–100.

Weber, R., Ghoshdastider, U., Spies, D., Duré, C., Valdivia-Francia, F., Forny, M., … others (2023). Monitoring the 5 utr landscape reveals isoform switches to drive translational efficiencies in cancer. *Oncogene*, *42*(9), 638–650.

Xie, J., Zhuang, Z., Gou, S., Zhang, Q., Wang, X., Lan, T., … others (2023). Precise genome editing of the kozak sequence enables bidirectional and quantitative modulation of protein translation to anticipated levels without affecting transcription. *Nucleic Acids Research*, *51*(18), 10075–10093.

Xu, C., & Zhang, J. (2020). Mammalian alternative translation initiation is mostly nonadaptive. *Molecular Biology and Evolution*, *37*(7), 2015–2028.

Yang, J., Cui, Y., Yu, D., Zhang, G., Cao, R., Gu, Z., … others (2023). A noncoding a-to-u kozak site change related to the high transmissibility of alpha, delta, and omicron vocs. *Molecular Biology and Evolution*, *40*(6), msad142.

Zhao, J., Li, Y., Wang, C., Zhang, H., Zhang, H., Jiang, B., … Song, X. (2020). Iresbase: a comprehensive database of experimentally validated internal ribosome entry sites. *Genomics, Proteomics and Bioinformatics*, *18*(2), 129–139.

Zheng, W., Fong, J. H., Wan, Y. K., Chu, A. H., Huang, Y., Wong, A. S., & Ho, J. W. (2023). Translation rate prediction and regulatory motif discovery with multi-task learning. In *International conference on research in computational molecular biology* (pp. 139–154).
